# Integrated bioinformatics and machine-learning screening for immune-related genes in diagnosing non-alcoholic fatty liver disease with ischemic stroke and RRS1 pan-cancer analysis

**DOI:** 10.3389/fimmu.2023.1113634

**Published:** 2023-04-05

**Authors:** Huayan Bao, Jianwen Li, Boyang Zhang, Ju Huang, Danke Su, Lidong Liu

**Affiliations:** ^1^ Department of Medical Imaging Center; Guangxi Key Clinical Specialty (Medical Imaging Department); Dominant Cultivation Discipline of Guangxi Medical University Cancer Hospital (Medical Imaging Department), Guangxi Medical University Cancer Hospital, Nanning, China; ^2^ Department of Neurosurgery, Guangxi Medical University Cancer Hospital, Nanning, China

**Keywords:** non-alcoholic fatty liver disease, ischemic stroke, machine learning, diagnosis, immune infiltration, pan-cancer

## Abstract

**Background:**

The occurrence of ischemic stroke (IS) is associated with nonalcoholic fatty liver disease (NAFLD). The cancer burden of NAFLD complicated by IS also warrants attention. This study aimed to identify candidate immune biomarkers linked to NAFLD and IS and analyze their association with cancer.

**Methods:**

Two of each of the NAFLD and IS datasets were downloaded, differentially expressed genes (DEGs) were identified, and module genes were screened *via* weighted gene coexpression network analysis (WGCNA). Subsequently, utilizing machine learning (least absolute shrinkage and selection operator regression, random forest and support vector machine-recursive feature elimination) and immune cell infiltration analysis, immune-related candidate biomarkers for NAFLD with IS were determined. Simultaneously, a nomogram was established, the diagnostic efficacy was assessed, and the role of candidate biomarkers in cancer was ascertained through pan-cancer analyses.

**Results:**

In this study, 117 and 98 DEGs were identified from the combined NAFLD and IS datasets, respectively, and 279 genes were obtained from the most significant modules of NAFLD. NAFLD module genes and IS DEGs were intersected to obtain nine genes, which were enriched in the inflammatory response and immune regulation. After overlapping the results of the three machine learning algorithms, six candidate genes were obtained, based on which a nomogram was constructed. The calibration curve demonstrated good accuracy, and the candidate genes had high diagnostic values. The genes were found to be related to the immune dysregulation of stroke, and *RRS1* was strongly associated with the prognosis, immune cell infiltration, microsatellite instability (MSI), and tumor mutation burden (TMB).

**Conclusion:**

Six common candidate immune-related genes (*PTGS2, FCGR1A, MMP9, VNN3, S100A12*, and *RRS1*) of NAFLD and IS were identified, and a nomogram for diagnosing NAFLD with IS was established. *RRS1* may serve as a candidate gene for predicting the prognosis of patients with cancer who have NAFLD complicated by IS, which could aid in their diagnosis and treatment.

## 1 Introduction

In the absence of a history of heavy alcohol consumption or another chronic liver disease, steatosis in >5% of hepatocytes is referred to as nonalcoholic fatty liver disease (NAFLD) (1). It is associated with the increased risk of various extrahepatic complications, including cardiovascular disease, type 2 diabetes, chronic kidney disease, and intrahepatic and extrahepatic malignancies (2, 3).

Recent studies have shown that NAFLD may be linked to a higher risk of ischemic stroke (IS) (4). IS, one of the main causes of disability and death, is a complex disease resulting from the interplay of environmental and genetic risk factors (5). According to a prospective study, NAFLD is an independent predictive factor for IS, and the more severe the NAFLD, the higher the incidence of IS (6).. A recent meta-analysis on the correlation between NAFLD and carotid atherosclerosis and IS established that progressive hepatic steatosis can significantly increase the probability of carotid atherosclerosis and stroke in patients with NAFLD (7). The pathophysiological mechanism of NAFLD leading to IS may include enhanced activation of the liver and systemic inflammatory response, oxidative stress, metabolic disorder, imbalance in adipokines, cytokines, etc., and progressive atherosclerosis (especially carotid atherosclerosis) (8–11). Likewise, these mechanism are also associated with the development and progression of cancer. With the accelerated aging process and the prevalence of unhealthy lifestyles, the cancer burden of NAFLD patients with IS also deserves attention.

According to reports, the onset and progression of NAFLD involve crosstalk or their temporal involvement among immune cells such as innate-like T cells, neutrophils, monocytes, B cells, and Dendritic cells (12). In addition, studies have shown that IS is also closely related to immune cell infiltration. Brain-resident immune cells (such as microglial, meningeal, and perivascular macrophages) and peripheral immune cells (such as neutrophils, macrophages, dendritic cells, lymphocytes, etc.) are involved in the development of IS (13). It is well known that disturbances in the immune microenvironment are also associated with the occurrence and development of cancer. The alteration in the immunological microenvironment is significant to the process of NAFLD leading to IS, but the particular molecular mechanism has yet to be confirmed.

In this study, two NAFLD and IS datasets were downloaded from the Gene Expression Omnibus (GEO, http://www.ncbi.nlm.nih.gov/geo) database. The “Limma” package was used to identify the differentially expressed genes (DEGs) in NAFLD and IS. Subsequently, weighted gene coexpression network analysis (WGCNA) was applied to determine the critical module in NAFLD. To discern the candidate immune-related biomarkers for NAFLD with IS, functional enrichment analysis, protein-protein interaction (PPI) network creation, application of machine-learning algorithms (least absolute shrinkage and selection operator [LASSO], random forest [RF], and support vector machine-recursive feature elimination [SVM-RFE]), evaluation of nomogram and receiver operating characteristic (ROC) curve, and analysis of immune cell infiltration were performed. Furthermore, pan-cancer analysis was conducted to assess the functions of biomarkers in cancer.

## 2 Materials and methods

### 2.1 Microarray data

**Figure 1:**
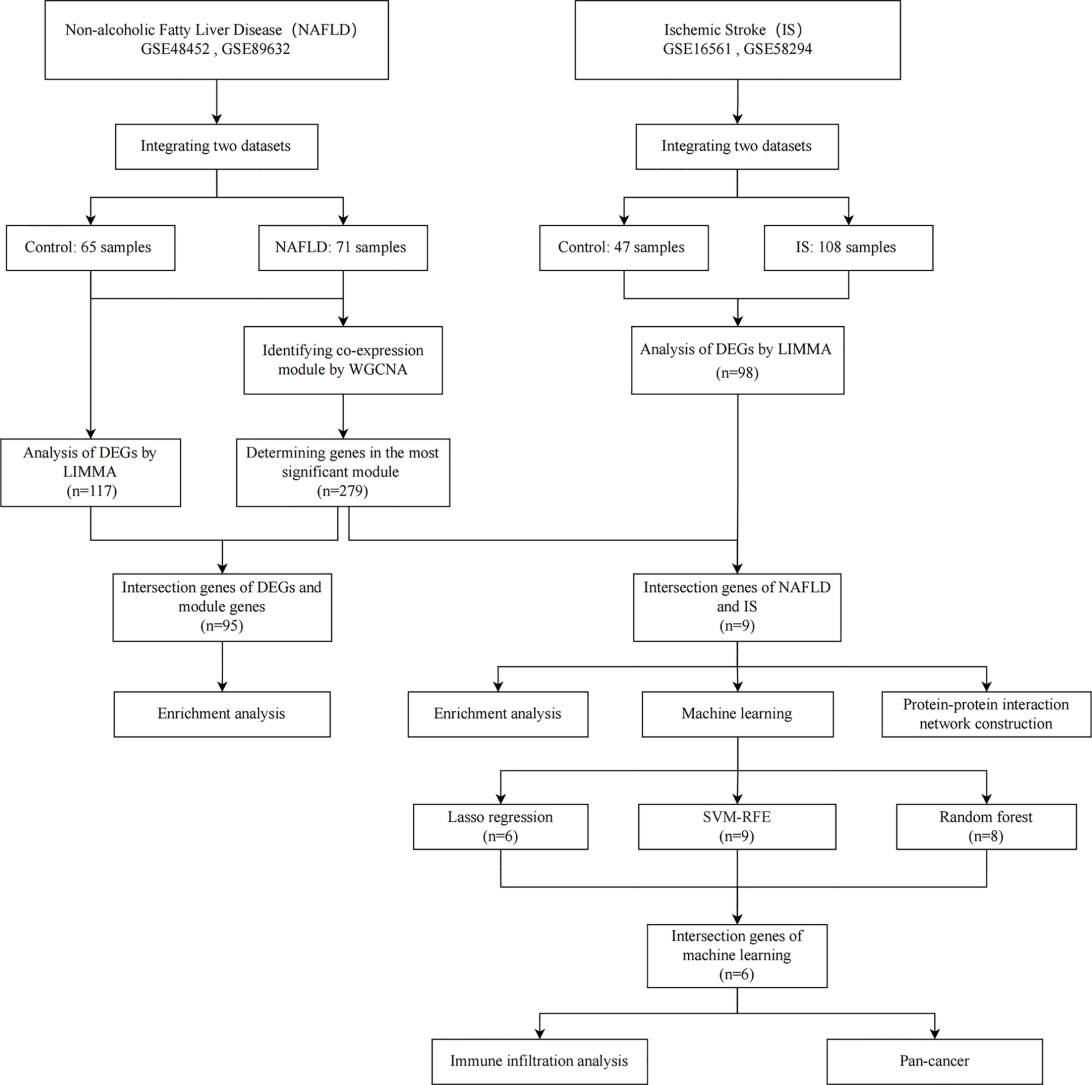
Depiction of the study flow chart.

The flowchart employed for this research is depicted in **Figure 1**. The 2 NAFLD datasets (GSE48452 and GSE89632) and 2 IS datasets (GSE16561 and GSE58294) were downloaded from the GEO database. The GSE48452 dataset (platform: GPL11532) includes 41 controls and 32 NAFLD patients. The GSE89632 dataset (platform: GPL14951) includes 24 controls and 39 NAFLD patients. The GSE16561 dataset (platform: GPL6883) includes 24 controls and 39 IS patients. The GSE58294 dataset (platform: GPL570) includes 23 controls and 69 IS patients.

### 2.2 Data preprocessing and selection of differentially expressed genes

The 4 original datasets were first subjected to background correction and then normalized by using the “affy” package in the R software. Subsequently, probe-level data were converted to their respective gene expression values. When a gene corresponds to multiple probes, the gene expression value is replaced with the average expression value. Following the integration of the datasets, the batch effect was eliminated by using the combat function of the “SVA” package in R, as there was a notable batch difference across datasets from different platforms. Finally, in order to identify DEGs between the disease and control groups, the “Limma” package in R software was used with the false discovery rate (FDR) of <0.05 and |log_2_ Fold change (FC)| >0.6 as the screening criterion.

### 2.3 Construction of co-expressed gene modules

The weighted co-expression network for the expression matrix of the NAFLD dataset was created using the “WGCNA” package in the R software. For further analysis, the genes with a median absolute deviation (MAD) >25% were selected. To ensure data integrity, the “goodSamplesGenes” function was applied. To determine and validate the optimal soft threshold (β) and construct a scale-free network, the “pickSoftThreshold” was used. Next, the adjacency matrix was turned into a topological overlap matrix (TOM), and the gene modules were constructed using hierarchical clustering and the dynamic pruning-tree algorithm. The gene significance (GS) and module significance (MS) were calculated by using phenotype and module data in order to examine the relationship between modules or genes and the clinical features.

### 2.4 Functional enrichment analysis and ppi network establishment

The “clusterProfiler” package in the R software, the functional enrichment analysis of the Gene Ontology (GO) and Kyoto Encyclopedia of Genes and Genomes (KEGG) was performed, and P < 0.05 was considered to indicate statistical significance. GO and KEGG analyses were performed in accordance with the intersection genes of the most significant module genes and DEGs of NAFLD, and the intersection genes of the most significant module genes of NAFLD and DEGs of IS, respectively. In addition, we also utilize the String database (http://string-db.org/) for Protein–Protein Interaction (PPI) network construction. The intersection genes of the most significant module genes of NAFLD and the DEGs of IS were imported into the String database, and then the species was selected as “Homo sapiens”, and finally it was considered significant when it was greater than the minimum interaction score of 0.4.

### 2.5 Machine learning for screening candidate genes

To identify the important biomarkers, candidate genes for the diagnosis of NAFLD with IS were further screened by using 3 machine-learning algorithms. LASSO regression, which can be used for variable selection and regularization to increase the prediction accuracy, was performed using the “glmnet” package in the R software. RFE of the RF algorithm is a supervised machine-learning algorithm that can be applied to rank the intersection genes of NAFLD and IS as well as to determine the genes with relative importance >2 as feature genes. SVM-RFE is an SVM-based machine-learning algorithm that can determine the most suitable subset of genes while avoiding overfitting. Finally, the genes selected by the three machine learning algorithms were intersected, and the intersected genes were used for subsequent analysis.

### 2.6 Establishment of nomogram and evaluation of predictive efficiency

The nomograms were constructed using the ‘rms’ package in R software based on the intersection genes screened by using three machine-learning algorithms. Then, the calibration curve was applied to evaluate the nomogram’s predictive capability. When establishing the ROC curve of the candidate genes, the area under the curve (AUC) was computed to assess the prediction accuracy of NAFLD with IS.

### 2.7 Immune cell infiltration analysis

Using the ssGSEA algorithm of the “GSVA” package in R software, the immune infiltration of IS was quantified. Spearman’s correlations were computed to investigate the correlation between immune-infiltrating cells and the intersection of genes of machine-learning algorithms.

### 2.8 Analysis of tumor-related prognosis

The “forestplot” package in R software was used to run univariate Cox regression analyses, after which the p-value, HR, and 95% CI values were computed. Subsequently, the data was visualized through a forest plot.

### 2.9 Analysis of tumor-related immune infiltration

TIMER, xCELL, MCPCOUNTER, CIBERSORT, EPIC, and QUANTISEQ algorithms were used for further exploring the correlation between candidate gene expression and immune infiltration in all TCGA tumors. Then, we looked at the relationship between candidate genes and the expression of genes relevant to immunological checkpoints in diverse cancers. Moreover, we examined the relationship between MSI or TMB in different cancers and the expression of candidate genes.

### 2.10 Statistical analysis

R software (version 4.2.0; https://www.r-project.org/) was utilized for all statistical analyses and graph generation. The Student t-test was utilized to compare group differences. The predictive performance of the candidate genes utilized to build the predictive model was evaluated using the ROC curve. P<0.05 was considered to indicate statistical significance.

## 3 Results

### 3.1 Identification of differentially expressed genes

**Figure 2:**
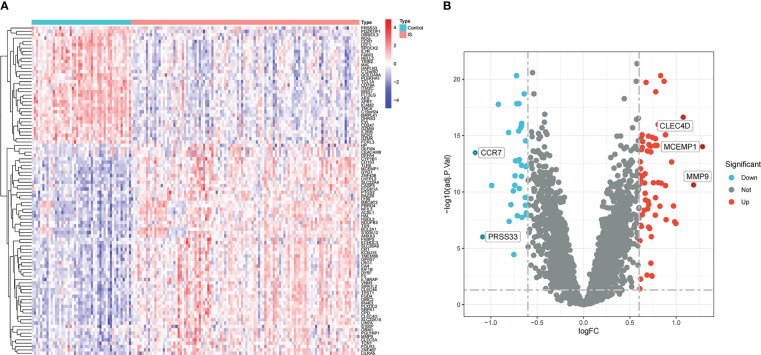
DEGs identification in the integrated IS dataset. **(A)** Rows represent DEGs, and each column refers to a sample. Red and blue represent upregulated and downregulated DEGs, respectively. **(B)** Red and green circles represent upregulated and downregulated DEGs, respectively.

**Figure 3:**
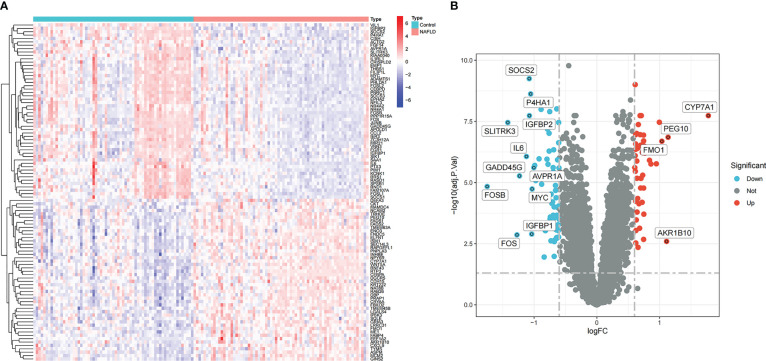
Identification of DEGs in the integrated NAFLD dataset. **(A)** Rows represent DEGs, and each column refers to a sample. Red and blue represent the upregulated and downregulated DEGs, respectively. **(B)** Red and green circles represent upregulated and downregulated DEGs, respectively.

In the comprehensive dataset of NAFLD, 117 DEGs were identified using the “Limma” package in R. A total of 98 DEGs were identified in the combined dataset of IS. Volcano maps and heatmaps were used to visualize the DEGs of IS and NAFLD, as shown in **Figures 2**, **3**, respectively. The detailed DEGs of NAFLD or IS are listed in **Supplementary Table S1** and **Supplementary Table S2**, respectively.

### 3.2 Construction of weighted gene coexpression network and identification of key modules

**Figure 4:**
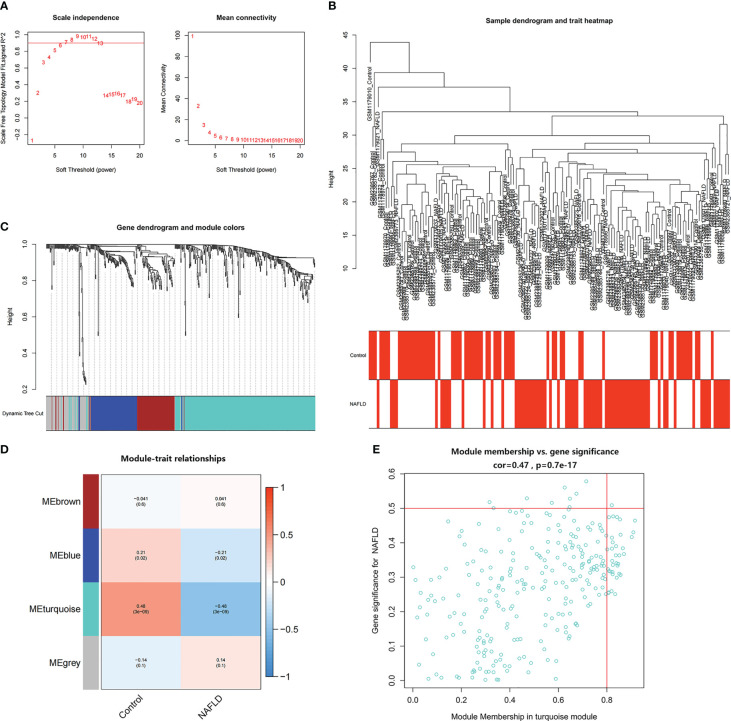
Identification of module genes in NAFLD. **(A)** The soft threshold (β) was determined to be 6 when the correlation coefficient was 0.9. **(B)** Clustering dendrogram of NAFLD and control samples. **(C)** Each branch in the cluster diagram refers to a gene, and different colors represent a gene co-expression module. **(D)** Heatmap of module–trait relationships. **(E)** Scatter plot of the correlation between gene module membership and gene significance in the turquoise module.

WGCNA was used to identify the most significantly related modules in the NAFLD combined dataset. The soft threshold was set at β = 6 (scale-free R^2^ = 0.9) to fit the gene expression associated with a scale-free network (**Figure 4A**). After removing the abnormal samples, the clustering dendrogram of NAFLD and controls was obtained, as shown in **Figure 4B**. Next, using dynamic hybrid shearing, four gene coexpression modules were produced (**Figure 4C**). The correlation of the gene modules with NAFLD and controls is depicted in **Figure 4D**, and the turquoise module (containing 279 genes) demonstrated the most significant correlation with NAFLD (correlation coefficient = 0.6, p = 3e-09). Additionally, in the turquoise module, a strong association was noted between module membership and GS (correlation coefficient = 0.47, p = 9.7e-17) (**Figure 4E**). Therefore, this module was used for subsequent analyses.

### 3.3 Functional enrichment analysis and construction of PPI network

**Figure 5:**
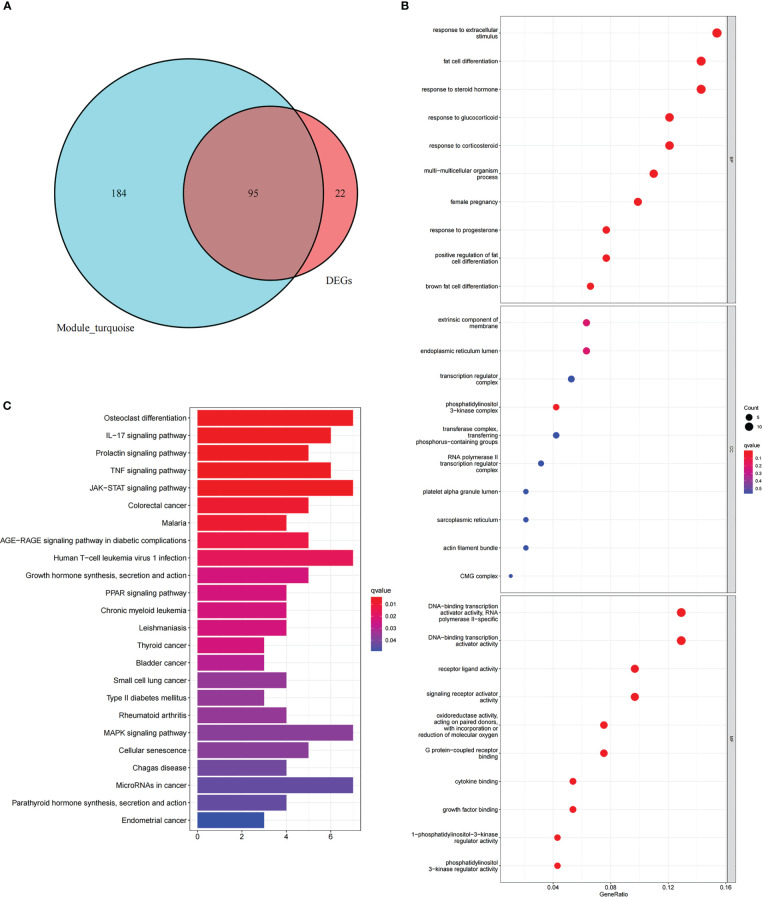
Functional enrichment analysis of intersection genes in NAFLD. **(A)** With reference to the Venn diagram, the turquoise module of NAFLD and the DEGs of NAFLD contain 95 intersection genes. **(B)** GO enrichment analysis of the intersecting genes. The X-axis represents the gene ratio for each term, while the Y-axis refers to the different terms in GO. The p-value is indicated as the color of the circle, and the circle size indicates the number of genes for the corresponding item. **(C)** The KEGG pathway enrichment analysis results of the intersecting genes.

First, the turquoise module genes of NAFLD with DEGs were overlapped, and 95 intersection genes were obtained using Venn diagrams (**Figure 5A**). Then, GO and KEGG functional enrichment analysis were employed to comprehend the biological functions of the intersection genes in NAFLD. GO enrichment analysis revealed that “response to extracellular stimulus,” “fat cell differentiation,” and “response to steroid hormone” were the mainly enriched biological process (BP) (**Figure 5B**). Cellular component (CC) analysis showed that the intersection genes were mainly enriched in “extrinsic component of membrane,” “endoplasmic reticulum lumen,” and “phosphatidylinositol 3-kinase complex” (**Figure 5B**). In terms of molecular function (MF), “DNA-binding transcription activator activity,” “receptor ligand activity,” and “signaling receptor activator activity” were the most important items (**Figure 5B**). According to KEGG enrichment analysis (**Figure 5C**), the intersecting genes were primarily enriched in the pathways of “IL-17 signaling pathway,” “TNF signaling pathway,” and “JAK-STAT signaling pathway.”

**Figure 6:**
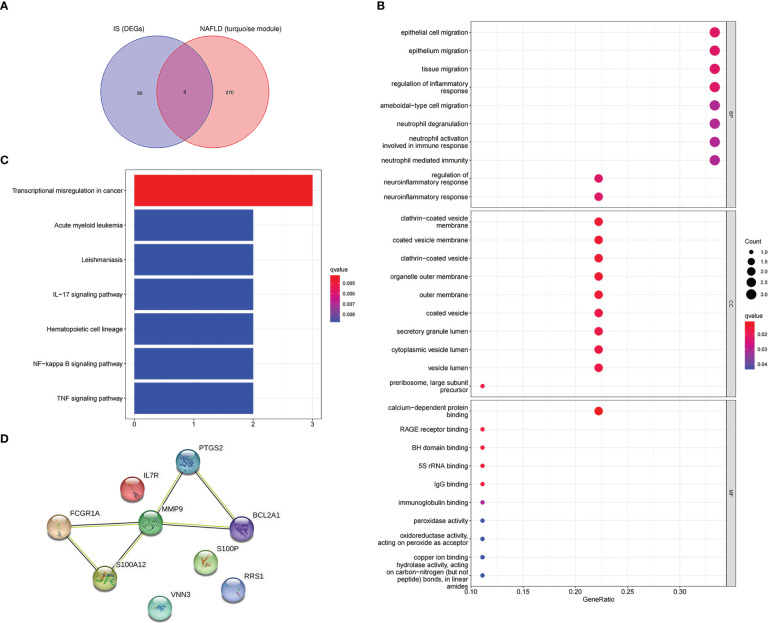
Enrichment analysis and the PPI network of the intersection genes of NAFLD and IS. **(A)** The Venn diagram depicts the 9 intersection genes in the turquoise module of NAFLD and the DEGs of IS. **(B)** GO enrichment analysis of 9 intersection genes. **(C)** KEGG analysis of 9 intersection genes. **(D)** The PPI network illustrates the interactions among the 9 intersection genes.

To further explore whether the key genes related to NAFLD were associated with the pathogenesis of IS, the turquoise module genes of NAFLD and the DEGs of IS were intersected to obtain nine intersection genes, which were displayed with Venn diagrams (**Figure 6A**). According to GO enrichment analysis, these intersection genes were primarily enriched in the BP of “epithelial cell migration,” “regulation of inflammatory response,” “neutrophil activation involved in immune response,” and “regulation of neuroinflammatory response”, CC of “clathrin-coated vesicle membrane,” “outer membrane,” and “secretory granule lumen,” and MF of “calcium-dependent protein binding,” “RAGE receptor binding,” and “IgG binding” (**Figure 6B**). According to KEGG enrichment analysis, the nine genes were primarily enriched in “transcriptional misregulation in cancer,” “IL-17 signaling pathway,” and “NF-kappa B signaling pathway” (**Figure 6C**). In summary, these nine common genes were highly related to immune regulation and inflammatory response and also associated with transcriptional dysregulation in cancer. The functional enrichment results of the common genes were similar to those of NAFLD, which implies that the common genes could be used for subsequent analysis.

In addition, after the identification of the nine intersection genes relevant to immunity and cancer, a PPI network was created to understand the interaction (**Figure 6D**).

### 3.4 Screening candidate genes with machine learning

**Figure 7:**
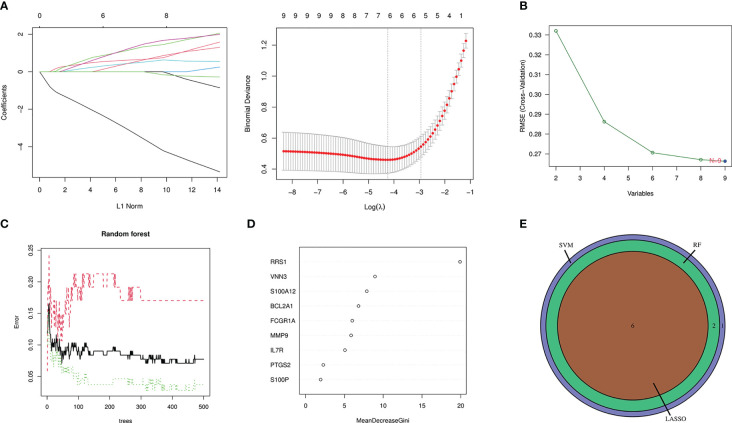
Machine-learning algorithm screening for candidate biomarkers of NAFLD with IS. **(A)** Biomarker screening in the Lasso regression. The lowest point of the curve corresponds to the optimal number of genes (n = 6). **(B)** SVM-RFE algorithm screening for biomarkers. **(C)** The relationship between the number of trees and the error rate is displayed in a random forest. **(D)** Ranking of genes according to their relative importance. **(E)** Venn diagram depicting 6 candidate genes screened by 3 machine-learning algorithms.

In this study, candidate genes were screened using the three machine learning algorithms of LASSO regression, SVM-RFE, and RF. First, LASSO regression analysis was applied for the intersection genes. After removing the redundant variables, six characteristic genes were identified as potential biomarkers (**Figure 7A**). Second, for the results of SVM-RFE analysis, when the eigengene was nine, the error of the classifier was the smallest (**Figure 7B**). Next, the relative importance of the genes was ranked using the RF algorithm, and eight characteristic genes were identified (**Figures 7C, D**). Finally, the genes analyzed with the three machine algorithms were intersected, and six candidate characteristic genes (*PTGS2*, *FCGR1A*, *MMP9*, *VNN3*, *S100A12*, and *RRS1*) were recognized (**Figure 7E**).

### 3.5 Diagnostic value evaluation

**Figure 8:**
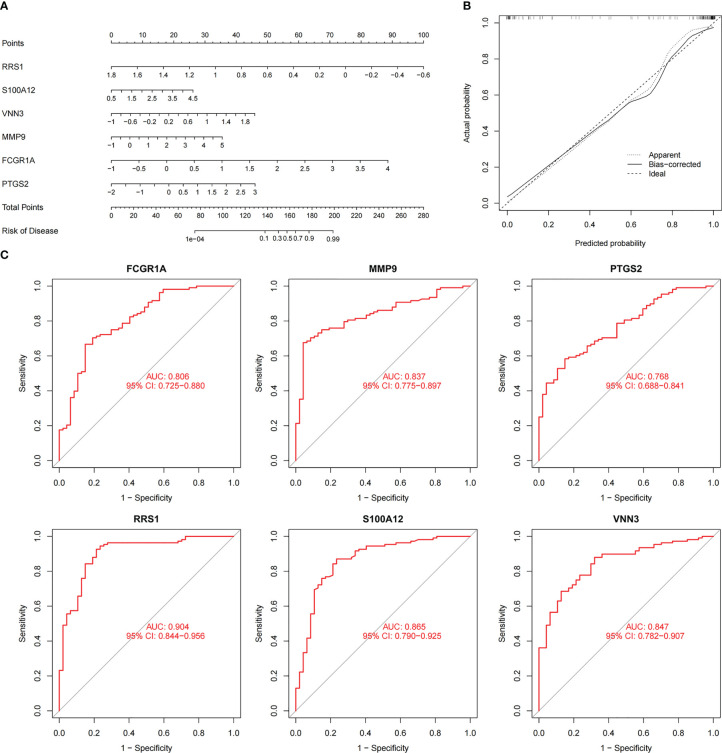
Nomogram construction and prediction accuracy evaluation. **(A)** Nomogram for diagnosing NAFLD with IS. **(B)** Calibration curves assessing the predictive accuracy of the nomograms. **(C)** ROC curves for each candidate gene (*PTGS2*, *FCGR1A*, *MMP9*, *VNN3*, *S100A12*, and *RRS1*).

To augment the clinical utility, a nomogram was constructed based on the six candidate genes (**Figure 8A**). The calibration curve revealed that the discrepancy between the actual and predicted values of IS risk was small, which demonstrated the high diagnostic value of the nomogram (**Figure 8B**). Moreover, ROC analysis was used to determine the AUC and 95% CI of each candidate gene. The findings were as follows: *FCGR1A* (AUC: 0.806, 95% CI: 0.725–0.880), *MMP9* (AUC: 0.837, 95% CI: 0.775–0.897), *PTGS2* (AUC: 0.768, 95% CI: 0.688–0.841), *RRS1* (AUC: 0.904, 95% CI: 0.844–0.956), *S100A12* (AUC: 0.865, 95% CI: 0.790–0.925), and *VNN3* (AUC: 0.847, 95% CI: 0.782–0.907) (**Figure 8C**). The above results show that all candidate genes have a high diagnostic value.

### 3.6 Analysis of immune cell infiltration

**Figure 9:**
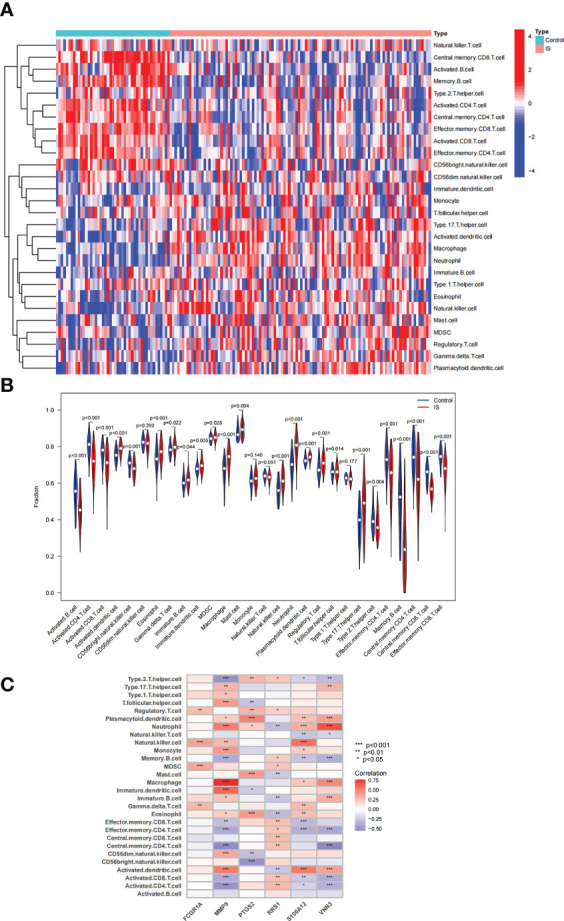
Analysis of immune cell infiltration associated with IS. **(A, B)** The distribution of 28 immune cells in IS and control samples is depicted *via* a heatmap and a violin plot. **(C)** Correlation between 6 candidate genes and immune cell infiltration.

The immune regulation mode in IS was further elucidated with immune cell infiltration analysis because the intersection genes of NAFLD and IS were primarily enriched in the inflammatory response and immune regulation. In the combined IS dataset, **Figure 9A** displays the percentage of immune cells in IS and controls. Compared with controls, lower levels of Effector.memory.CD8.T.cell, Type.2.T.helper.cell, Activated.B.cell, Activated.CD4.T.cell, Effector.memory.CD4.T.cell, Central.memory.CD8.T.cell, Activated.CD8.T.cell, CD56bright.natural.killer.cell, Memory.B.cell, and Central.memory.CD4.T.cell were observed in patients with IS. On the contrary, higher levels of Eosinophil, MDSC, Activated.dendritic.cell, Macrophage, Plasmacytoid.dendritic.cell, Gamma.delta.T.cell, Immature.B.cell, Mast.cell, Neutrophil, Regulatory.T.cell, Immature.dendritic.cell, Natural.killer.cell, and Type.17.T.helper.cell were noted (**Figure 9B**). Additionally, a correlation heatmap was used to demonstrate the correlation between the six candidate genes and immune cells (**Figure 9C**). The detailed results of correlation analysis between immune cells and candidate genes are listed in **Supplementary Table S3**.

### 3.7 Pan-cancer analysis of *RRS1* expression

**Figure 10:**
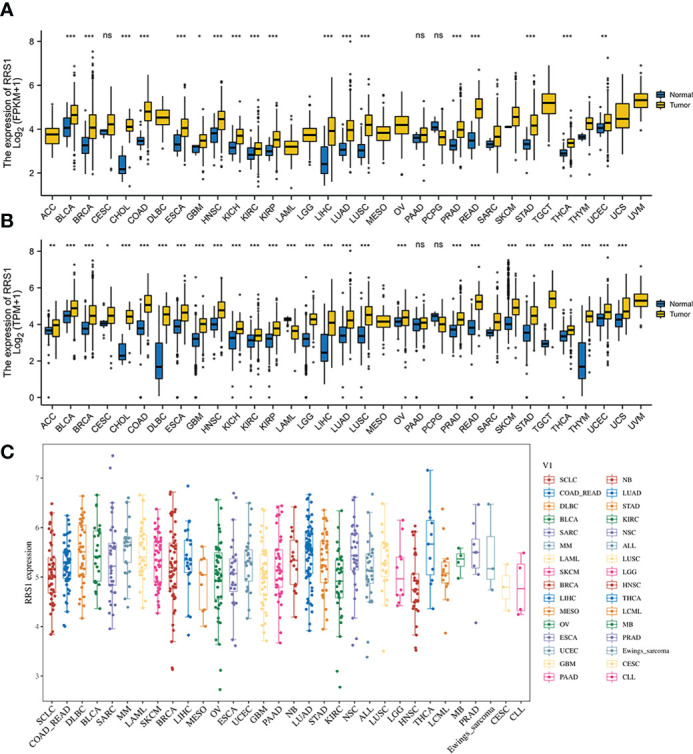
The expression of *RRS1* in pan-cancer. **(A)** In the TCGA database, the expression of *RRS1* in diverse cancers. **(B)** In the TCGA and GTEx databases, the expression of *RRS1* in diverse cancers. **(C)** *RRS1* expression in different cell lines. *p < 0.05, **p < 0.01, ***p <0.001. ns, no significance.

According to the KEGG enrichment assessment, the intersection genes of NAFLD and IS were associated with cancer transcriptional dysregulation. In addition, *RRS1* showed the highest diagnostic value for NAFLD with IS (AUC: 0.904, 95% CI: 0.844–0.956). Therefore, the immune-related gene *RRS1* was selected for further pan-cancer analysis. In the TCGA database, *RRS1* was highly expressed in 18 cancer types, including CHOL, BRCA, and BLCA, with a statistical significance (**Figure 10A**). The data for normal tissue in the GTEx database were then collected, and *RRS1* was found to be weakly expressed only in LAML but strongly expressed in 27 cancers, including ACC, BLCA, and BRCA (**Figure 10B**). The expression status of *RRS1* in each tumor cell line is depicted in **Figure 10C**.

### 3.8 Prognostic value of *RRS1* in pan-cancer analysis

**Figure 11:**
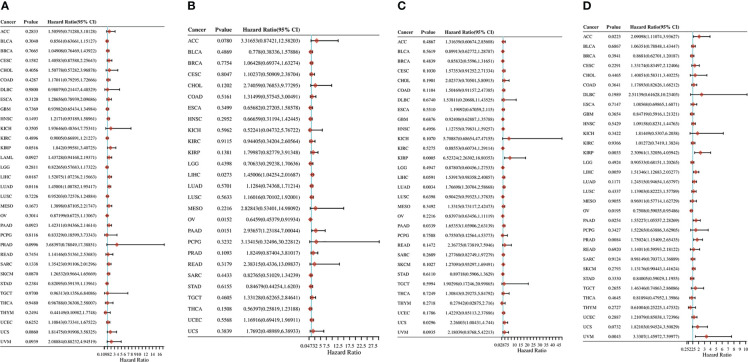
Pan-cancer analysis of the correlation between *RRS1* expression and prognosis. **(A)** The correlation between *RRS1* expression and OS in diverse cancers. **(B)** The correlation between *RRS1* expression and DFS in diverse cancers. **(C)** The correlation between *RRS1* expression and DSS in diverse cancers. **(D)** The correlation between *RRS1* expression and PFS in diverse cancers.

The relationship between the expression level of *RRS1* and the patient prognosis was investigated by determining progression-free survival (PFS), disease-free survival (DFS), disease-specific survival (DSS), and overall survival (OS). *RRS1* expression was substantially linked to LIHC and LUAD in OS analysis and acted as a risk factor in both LIHC and LUAD (**Figure 11A**). The DFS study found a significant relationship among three cancers—OV, LIHC, and PAAD—and *RRS1* expression (**Figure 11B**). Furthermore, *RRS1* was a protective factor in OV but a risk factor in LIHC and PAAD. In DSS analysis, the expression of *RRS1* was significantly associated with four cancers, namely, KIRP, PAAD, LUAD, and UCS, and was a risk factor in all four cancers (**Figure 11C**). PFS analysis indicated that the expression of *RRS1* was correlated with the PFS of seven malignancies, namely, ACC, KIRP, LIHC, OV, PAAD, PRAD, and UVM (**Figure 11D**). *RRS1* served as a protective factor only in OV and was a risk factor in the remaining six malignancies.

### 3.9 Immune cell infiltration related to *RRS1* in pan-cancer analysis

**Figure 12:**
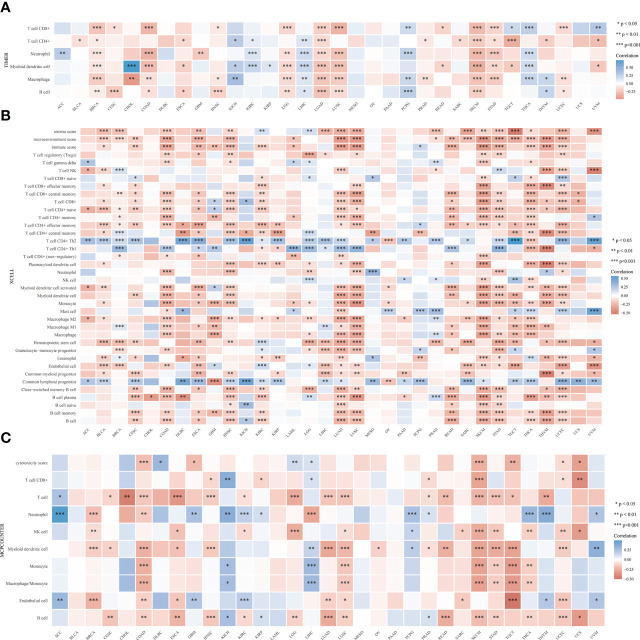
Pan-cancer analysis of the correlation between *RRS1* expression and immune cell infiltration. **(A)** Correlation between *RRS1* expression and the infiltration levels of various immune cells based on TIMER. **(B)** Correlation between *RRS1* expression and infiltration levels of various immune cells based on xCELLs. **(C)** Correlation between *RRS1* expression and infiltration levels of various immune cells based on MCPCOUNTER.

**Figure 13:**
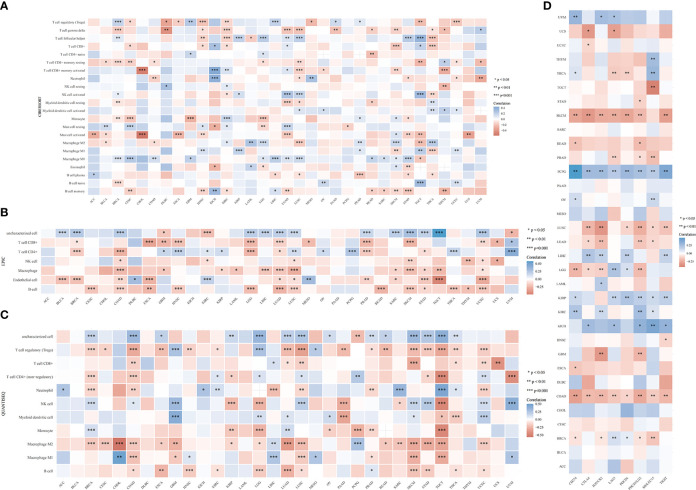
*RRS1* expression in relation to immune cell infiltration or immune-checkpoint genes analyzed *via* pan-cancer analysis. **(A)** Correlation between *RRS1* expression and infiltration levels of various immune cells based on CIBERSORT. **(B)** Correlation between the *RRS1* expression and infiltration levels of various immune cells based on EPIC. **(C)** Correlation between *RRS1* expression and infiltration levels of various immune cells based on QUANTISEQ. **(D)** *RRS1* expression and immune checkpoint genes correlation analyses.

The TIMER algorithm was used to assess the relationship between *RRS1* expression and the degree of immune cell infiltration in various malignancies. *RRS1* was related to B cells in 13 cancers, CD4+ T cells in 14 cancers, CD8+ T cells in 15 cancers, myeloid dendritic cells in 17 cancers, macrophages in 16 cancers, and neutrophils in 13 cancers according to TIMER analysis (**Figure 12A**). Moreover, *RRS1* was significantly negatively correlated with immune cells in LGG, COAD, LUSC, BRCA, SKCM, LUAD, and STAD but significantly positively correlated with immune cells in LIHC, PCPG, and THCA. In addition, the xCELL algorithm (**Figure 12B**), MCPCOUNTER program (**Figure 12C**), CIBERSORT algorithm (**Figure 13A**), EPIC algorithm (**Figure 13B**), and QUANTISEQ algorithm (**Figure 13C**) demonstrated that diverse immune cells were highly associated with *RRS1* expression. Furthermore, the expression levels in different malignancies were highly linked to immune checkpoint-related genes (**Figure 13D**). *RRS1* showed a positive correlation in KICH, PCPG, and KIRP, whereas it showed a negative correlation in SKCM, LUSC, and COAD.

### 3.10 MSI and TMB analysis

**Figure 14:**
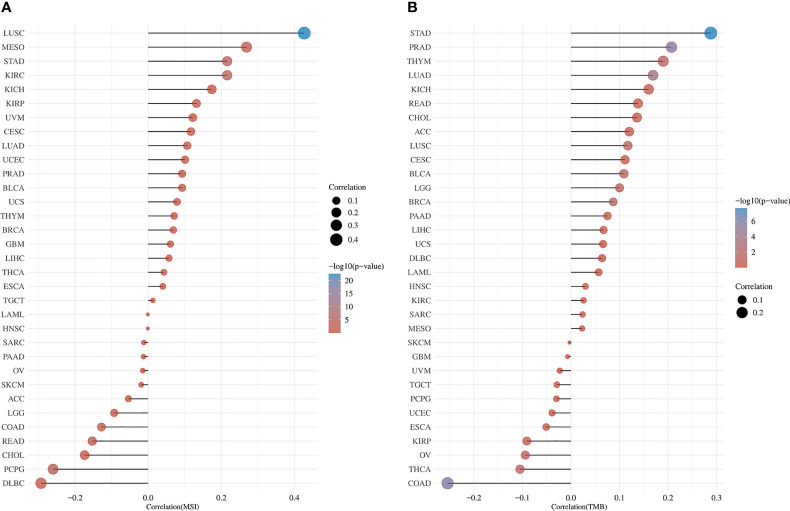
Pan-cancer analysis of *RRS1* expression in association with MSI or TMB. **(A)** *RRS1* expression and MSI correlation analyses. **(B)** *RRS1* expression and TMB correlation analyses.

MSI and TMB are expected to guide tumor immunotherapy. According to the results of MSI analysis, *RRS1* was significantly correlated with LUSC, MESO, DLBC, and PCBG (**Figure 14A**). In the TMB study, *RRS1* was significantly associated with STAD, PRAD, LUAD, and COAD (**Figure 14B**).

## 4 Discussion

NAFLD, a disease closely associated with metabolic dysfunction, has become the most common cause of chronic liver disease worldwide (14). NAFLD, together with the high incidence of hepatic (cirrhosis, liver cancer, etc.) and extrahepatic (including type 2 diabetes, myocardial infarction, IS, and extrahepatic tumors) complications, has imposed a huge burden on public health and the economy (2, 3, 15–17). IS is one of the main causes of disability and death, but the pathophysiological mechanisms of NAFLD and IS have not been completely elucidated. Moreover, the cancer burden of NAFLD complicated by IS warrants attention. In this study, six common immune-related candidate genes (*PTGS2*, *FCGR1A*, *MMP9*, *VNN3*, *S100A12*, and *RRS1*) for NAFLD and IS were identified *via* comprehensive bioinformatic analysis and machine learning algorithms.

Atherosclerosis, which can cause blood flow restriction and potential plaque rupture risk, is one of the causes of IS (18, 19). PTGS2, also known as COX-2, is upregulated in the macrophages of atherosclerotic lesions and may augment the inflammatory response in atherosclerosis (20, 21). S100A12 is connected with NAFLD-related diseases (including obesity, type 2 diabetes, and inflammation) and may participate in the development of atherosclerosis by mediating the pathophysiological processes of vascular inflammation, calcification, and oxidative stress (22–24). Dysregulation of MMP-9 plays a pertinent role in various pathophysiological processes, such as inflammation, atherosclerosis, central nervous system diseases, and autoimmune diseases (25–28). Importantly, elevated serum MMP-9 levels are associated with poor prognosis in IS (29, 30). Additionally, many studies have confirmed that PTGS2, S100A12, and MMP-9 gene polymorphisms are linked to a high risk of IS (31–35).

Previous studies have demonstrated that inflammatory response and immune regulation are involved in the development of IS (11, 36). Within hours after the onset of IS, the neutrophil count in the peripheral blood increases exponentially (37). Neutrophils accumulate in the brain after the onset of IS and release toxic signals, such as neutrophil extracellular traps, which prevent vascular reconstruction and repair after IS (38). Simultaneously, peripheral blood monocyte count increases significantly within 16 days after the onset of IS (39). In the early stages of IS, M1 macrophages can disrupt the integrity of the blood-brain barrier and contribute to the resolution of inflammation after switching to the M2 phenotype (40). In the present study, patients with IS exhibited higher levels of Eosinophil, MDSC, Activated.dendritic.cell, Macrophage, Plasmacytoid.dendritic.cell, Type.17.T.helper.cell, Immature.B.cell, Neutrophil, Gamma.delta.T.cell, Natural.killer.cell, Mast.cell, Immature.dendritic.cell, and Regulatory.T.cell, which agrees with previous studies.

Furthermore, a relationship was observed between the levels of various immune cells and the expressions of candidate genes. MMP-9 is one of the widely studied members of matrix metalloproteinases (MMPs) (41). In inflamed tissues, MMPs can be produced by various immune cells, such as neutrophils and macrophages (27, 42, 43). The level of MMP-9 in patients with nonalcoholic steatohepatitis (NASH) is significantly higher than that in patients with hepatitis C, and the enzyme is mainly localized in neutrophils in the liver tissues of patients with NASH (44). S100A12 is predominantly secreted by neutrophils and monocytes and plays a crucial role in inflammatory disorders (45, 46). Furthermore, elevated levels of neutrophil-to-lymphocyte ratio (NLR), which reflects systemic inflammation, affect the severity and prognosis of cardiovascular diseases, and VNN3 was confirmed to be independently associated with NLR (47). In summary, the candidate genes share a close relationship with inflammatory response and immune regulation, which establishes their significance in the immunological dysregulation process in IS.

As the KEGG enrichment analysis results revealed that the intersection genes of NAFLD and IS were associated with cancer, the effect of *RRS1* (the gene with the greatest prediction performance of NAFLD with IS) was further investigated in pan-cancer. In TCGA and GTEx databases, the differential expression analysis of *RRS1* in 28 tumor types, including BRCA, LIHC, and STAD, showed statistical significance. Moreover, the findings of this research signified that *RRS1* is a prognostic risk factor in various cancers. These results indicate that *RRS1* can promote the development of tumors and may be a potential marker for poor prognosis. *RRS1* can promote the development of HCC by enhancing ribosome biogenesis and attenuating RPL11-MDM2-P53 signaling (48). Furthermore, *RRS1* may augment breast cancer cell invasion and metastasis *via* the RPL11-c-Myc-SNAIL axis (49). Also, the downregulation of lncRNA SET-binding factor 2-antisense RNA1 can upregulate miR-143 and inhibit *RRS1* and ultimately restrict the progression of breast cancer (50). In addition, dysregulation of *RRS1* is involved in the development of several malignancies and the progression of various tumors, including papillary thyroid carcinoma, retinoblastoma, and neuroblastoma (51–54). These studies confirm the key role of *RRS1* in tumor progression. Notably, we observed that *RRS1* was closely related to CD8+ T cells, neutrophils, and other immune cells. *RRS1* was significantly negatively correlated with immune infiltrating cells in tumors, such as COAD, LUAD, LUSC, and SKCM. Moreover, our study identified that *RRS1* is closely associated with immune checkpoint-related genes, MSI, and TMB of various tumors. Previous studies have shown that both MSI and TMB are associated with the efficacy of immunotherapy (55). The dysregulation of *RRS1* may affect the efficacy of immunotherapy in patients, which remains to be confirmed by further studies. In brief, the expression of *RRS1* affects the prognosis of many tumors and is related to immune regulation. *RRS1* may be a candidate gene for predicting poor prognosis in patients with cancer who have NAFLD complicated by IS.

This study has several limitations. First, although the two separate datasets of NAFLD and IS were merged, the total sample size remained small. Second, the immune-related candidate genes identified in this study have not yet been experimentally verified. However, previous preclinical and clinical studies have confirmed the potential correlation of the candidate genes with NAFLD and IS, which supports the reliability of this study. Third, the results should be confirmed using the data in the database in subsequent clinical studies that integrate the clinical information of patients with NAFLD and IS, such as sex and age.

## 5 Conclusion

Six common immune-related genes (*PTGS2*, *FCGR1A*, *MMP9*, *VNN3*, *S100A12*, and *RRS1*) associated with NAFLD and IS were identified using bioinformatics methods and machine learning algorithms, and a diagnostic nomogram of NAFLD patients with IS was established. Meanwhile, we pointed out that dysregulation of *RRS1* may be a high risk factor for cancer in patients with NAFLD complicated by IS. Further research on *RRS1* is expected to aid in the diagnosis and treatment of this type of cancer patient.

## Data availability statement

The datasets presented in this study can be found in online repositories. The names of the repository/repositories and accession number(s) can be found in the article/**Supplementary Material**.

## Author contributions

HB and BZ contributed to data analysis and manuscript drafting. JL contributed to data analysis and revised the manuscript. JH participated in data collection and figure editing. DS and LL conceived the study and revised the manuscript. All authors contributed to the article and approved the submitted version.

## References

[1] Powell EE Wong VWS Rinella M. Non-alcoholic fatty liver disease. Lancet (Lond Engl) (2021) 397(10290):2212–24. doi: 10.1016/s0140-6736(20)32511-3 33894145

[2] Targher G Tilg H Byrne CD. Non-alcoholic fatty liver disease: A multisystem disease requiring a multidisciplinary and holistic approach. Lancet Gastroenterol Hepatol (2021) 6(7):578–88. doi: 10.1016/s2468-1253(21)00020-0 33961787

[3] Kumar R Priyadarshi RN Anand U. Non-alcoholic fatty liver disease: Growing burden, adverse outcomes and associations. J Clin Trans Hepatol (2020) 8(1):76–86. doi: 10.14218/jcth.2019.00051 PMC7132013 32274348

[4] Alon L Corica B Raparelli V Cangemi R Basili S Proietti M. Risk of cardiovascular events in patients with non-alcoholic fatty liver disease: A systematic review and meta-analysis. Eur J Prev Cardiol (2022) 29(6):938–46. doi: 10.1093/eurjpc/zwab212 34939092

[5] Liu R Song P Gu X Liang W Sun W Hua Q. Comprehensive landscape of immune infiltration and aberrant pathway activation in ischemic stroke. Front Immunol (2021) 12:766724. doi: 10.3389/fimmu.2021.766724 35140708 PMC8818702

[6] Xu J Dai L Zhang Y Wang A Li H Wang Y. Severity of nonalcoholic fatty liver disease and risk of future ischemic stroke events. Stroke (2021) 52(1):103–10. doi: 10.1161/strokeaha.120.030433 33272125

[7] Tang ASP Chan KE Quek J Xiao J Tay P Teng M. Non-alcoholic fatty liver disease increases risk of carotid atherosclerosis and ischemic stroke: An updated meta-analysis with 135,602 individuals. Clin Mol Hepatol (2022) 28(3):483–96. doi: 10.3350/cmh.2021.0406 PMC9293613 35232007

[8] Duell PB Welty FK Miller M Chait A Hammond G Ahmad Z. Nonalcoholic fatty liver disease and cardiovascular risk: A scientific statement from the American heart association. Arteriosclerosis thrombosis Vasc Biol (2022) 42(6):e168–e85. doi: 10.1161/atv.0000000000000153 35418240

[9] Khanna S Parikh NS VanWagner LB. Fatty liver and cerebrovascular disease: Plausible association and possible mechanisms. Curr Opin Lipidology (2022) 33(1):31–8. doi: 10.1097/mol.000000000000079 PMC8702469 34799486

[10] Kasper P Martin A Lang S Kütting F Goeser T Demir M. Nafld and cardiovascular diseases: A clinical review. Clin Res cardiology: Off J German Cardiac Soc (2021) 110(7):921–37. doi: 10.1007/s00392-020-01709-7 PMC8238775 32696080

[11] Zera KA Buckwalter MS. The local and peripheral immune responses to stroke: Implications for therapeutic development. Neurotherapeutics: J Am Soc Exp Neurother (2020) 17(2):414–35. doi: 10.1007/s13311-020-00844-3 PMC7283378 32193840

[12] Huby T Gautier EL. Immune cell-mediated features of non-alcoholic steatohepatitis. Nat Rev Immunol (2022) 22(7):429–43. doi: 10.1038/s41577-021-00639-3 PMC8570243 34741169

[13] Iadecola C Buckwalter MS Anrather J. Immune responses to stroke: Mechanisms, modulation, and therapeutic potential. J Clin Invest (2020) 130(6):2777–88. doi: 10.1172/jci135530 PMC7260029 32391806

[14] Younossi ZM Koenig AB Abdelatif D Fazel Y Henry L Wymer M. Global epidemiology of nonalcoholic fatty liver disease-Meta-Analytic assessment of prevalence, incidence, and outcomes. Hepatol (Baltimore Md) (2016) 64(1):73–84. doi: 10.1002/hep.28431 26707365

[15] Paik JM Golabi P Younossi Y Mishra A Younossi ZM. Changes in the global burden of chronic liver diseases from 2012 to 2017: The growing impact of nafld. Hepatol (Baltimore Md) (2020) 72(5):1605–16. doi: 10.1002/hep.31173 32043613

[16] Mantovani A Csermely A Petracca G Beatrice G Corey KE Simon TG. Non-alcoholic fatty liver disease and risk of fatal and non-fatal cardiovascular events: An updated systematic review and meta-analysis. Lancet Gastroenterol Hepatol (2021) 6(11):903–13. doi: 10.1016/s2468-1253(21)00308-3 34555346

[17] Chen B Tang WHW Rodriguez M Corey KE Sanyal AJ Kamath PS. Nafld in cardiovascular diseases: A contributor or comorbidity? Semin Liver Dis (2022) 42(4):465–74. doi: 10.1055/s-0042-1757712 36241194

[18] Rahman MS Woollard K. Atherosclerosis. Adv Exp Med Biol (2017) 1003:121–44. doi: 10.1007/978-3-319-57613-8_7 28667557

[19] Donnan GA Fisher M Macleod M Davis SM. Stroke. Lancet (London England) (2008) 371(9624):1612–23. doi: 10.1016/s0140-6736(08)60694-7 18468545

[20] Linton MF Fazio S. Cyclooxygenase-2 and inflammation in atherosclerosis. Curr Opin Pharmacol (2004) 4(2):116–23. doi: 10.1016/j.coph.2003.12.003 15063354

[21] Mozos I Malainer C Horbańczuk J Gug C Stoian D Luca CT. Inflammatory markers for arterial stiffness in cardiovascular diseases. Front Immunol (2017) 8:1058. doi: 10.3389/fimmu.2017.01058 28912780 PMC5583158

[22] Oesterle A Bowman MA. S100a12 and the S100/Calgranulins: Emerging biomarkers for atherosclerosis and possibly therapeutic targets. Arteriosclerosis Thrombosis Vasc Biol (2015) 35(12):2496–507. doi: 10.1161/atvbaha.115.302072 PMC4771489 26515415

[23] Xiao X Yang C Qu SL Shao YD Zhou CY Chao R. S100 proteins in atherosclerosis. Clinica chimica acta; Int J Clin Chem (2020) 502:293–304. doi: 10.1016/j.cca.2019.11.019 31794767

[24] Delangre E Oppliger E Berkcan S Gjorgjieva M Correia de Sousa M Foti M. S100 proteins in fatty liver disease and hepatocellular carcinoma. Int J Mol Sci (2022) 23(19). doi: 10.3390/ijms231911030 PMC9570375 36232334

[25] Okazaki I Noro T Tsutsui N Yamanouchi E Kuroda H Nakano M. Fibrogenesis and carcinogenesis in nonalcoholic steatohepatitis (Nash): Involvement of matrix metalloproteinases (Mmps) and tissue inhibitors of metalloproteinase (Timps). Cancers (2014) 6(3):1220–55. doi: 10.3390/cancers6031220 PMC4190539 24978432

[26] Zhong C Yang J Xu T Xu T Peng Y Wang A. Serum matrix metalloproteinase-9 levels and prognosis of acute ischemic stroke. Neurology (2017) 89(8):805–12. doi: 10.1212/wnl.0000000000004257 PMC5580861 28747453

[27] Vandooren J Van den Steen PE Opdenakker G. Biochemistry and molecular biology of gelatinase b or matrix metalloproteinase-9 (Mmp-9): The next decade. Crit Rev Biochem Mol Biol (2013) 48(3):222–72. doi: 10.3109/10409238.2013.770819 23547785

[28] Ram M Sherer Y Shoenfeld Y. Matrix metalloproteinase-9 and autoimmune diseases. J Clin Immunol (2006) 26(4):299–307. doi: 10.1007/s10875-006-9022-6 16652230

[29] Tan C Liu Y Li W Deng F Liu X Wang X. Associations of matrix metalloproteinase-9 and monocyte chemoattractant protein-1 concentrations with carotid atherosclerosis, based on measurements of plaque and intima-media thickness. Atherosclerosis (2014) 232(1):199–203. doi: 10.1016/j.atherosclerosis.2013.11.040 24401238

[30] Li Y Han X Luo S Huang H Huang X Li M. Predictive value of longitudinal changes of serum matrix metalloproteinase-9 and brain-derived neurotrophic factor in acute ischemic stroke. Front Aging Neurosci (2022) 14:952038. doi: 10.3389/fnagi.2022.952038 36092813 PMC9452807

[31] Yi X Lin J Luo H Wang C Liu Y. Genetic variants of Ptgs2, Txa2r and Txas1 are associated with carotid plaque vulnerability, platelet activation and Txa2 levels in ischemic stroke patients. PloS One (2017) 12(7):e0180704. doi: 10.1371/journal.pone.0180704 28704403 PMC5507514

[32] Chen GZ Shan XY Cheng GP Tao HM. Cyclooxygenase-2 genetic polymorphism and stroke subtypes in Chinese. J Mol neuroscience: MN (2013) 51(2):467–73. doi: 10.1007/s12031-013-0078-5 23907768

[33] Chen L Chen X Wang Y Li S Huang S Wu Z. Polymorphisms of calgranulin genes and ischemic stroke in a Chinese population. J Inflamm Res (2022) 15:3355–68. doi: 10.2147/jir.S360775 PMC9191198 35706528

[34] Buraczynska K Kurzepa J Ksiazek A Buraczynska M Rejdak K. Matrix metalloproteinase-9 (Mmp-9) gene polymorphism in stroke patients. Neuromolecular Med (2015) 17(4):385–90. doi: 10.1007/s12017-015-8367-5 PMC4643105 26330106

[35] Misra S Talwar P Kumar A Kumar P Sagar R Vibha D. Association between matrix metalloproteinase family gene polymorphisms and risk of ischemic stroke: A systematic review and meta-analysis of 29 studies. Gene (2018) 672:180–94. doi: 10.1016/j.gene.2018.06.027 29906531

[36] Simats A Liesz A. Systemic inflammation after stroke: Implications for post-stroke comorbidities. EMBO Mol Med (2022) 14(9):e16269. doi: 10.15252/emmm.202216269 35971650 PMC9449596

[37] Gill D Sivakumaran P Aravind A Tank A Dosh R Veltkamp R. Temporal trends in the levels of peripherally circulating leukocyte subtypes in the hours after ischemic stroke. J stroke cerebrovascular diseases: Off J Natl Stroke Assoc (2018) 27(1):198–202. doi: 10.1016/j.jstrokecerebrovasdis.2017.08.023 28927686

[38] Kang L Yu H Yang X Zhu Y Bai X Wang R. Neutrophil extracellular traps released by neutrophils impair revascularization and vascular remodeling after stroke. Nat Commun (2020) 11(1):2488. doi: 10.1038/s41467-020-16191-y 32427863 PMC7237502

[39] Ma Y Yang S He Q Zhang D Chang J. The role of immune cells in post-stroke angiogenesis and neuronal remodeling: The known and the unknown. Front Immunol (2021) 12:784098. doi: 10.3389/fimmu.2021.784098 34975872 PMC8716409

[40] Qiu YM Zhang CL Chen AQ Wang HL Zhou YF Li YN. Immune cells in the bbb disruption after acute ischemic stroke: Targets for immune therapy? Front Immunol (2021) 12:678744. doi: 10.3389/fimmu.2021.678744 34248961 PMC8260997

[41] Li T Li X Feng Y Dong G Wang Y Yang J. The role of matrix metalloproteinase-9 in atherosclerotic plaque instability. Mediators Inflamm (2020) 2020:3872367. doi: 10.1155/2020/3872367 33082709 PMC7557896

[42] Parks WC Wilson CL López-Boado YS. Matrix metalloproteinases as modulators of inflammation and innate immunity. Nat Rev Immunol (2004) 4(8):617–29. doi: 10.1038/nri1418 15286728

[43] Olejarz W Łacheta D Kubiak-Tomaszewska G. Matrix metalloproteinases as biomarkers of atherosclerotic plaque instability. Int J Mol Sci (2020) 21(11). doi: 10.3390/ijms21113946 PMC7313469 32486345

[44] D’Amico F Consolo M Amoroso A Skarmoutsou E Mauceri B Stivala F. Liver immunolocalization and plasma levels of mmp-9 in non-alcoholic steatohepatitis (Nash) and hepatitis c infection. Acta Histochemica (2010) 112(5):474–81. doi: 10.1016/j.acthis.2009.05.005 19604544

[45] Pietzsch J Hoppmann S. Human S100a12: A novel key player in inflammation? Amino Acids (2009) 36(3):381–9. doi: 10.1007/s00726-008-0097-7 18443896

[46] Farokhzadian J Mangolian Shahrbabaki P Bagheri V. S100a12-Cd36 axis: A novel player in the pathogenesis of atherosclerosis? Cytokine (2019) 122:154104. doi: 10.1016/j.cyto.2017.07.010 28756107

[47] Wan G Ji L Xia W Cheng L Zhang Y. Screening genes associated with elevated Neutrophil−to−Lymphocyte ratio in chronic heart failure. Mol Med Rep (2018) 18(2):1415–22. doi: 10.3892/mmr.2018.9132 PMC6072186 29901123

[48] Cao P Yang A Li P Xia X Han Y Zhou G. Genomic gain of Rrs1 promotes hepatocellular carcinoma through reducing the Rpl11-Mdm2-P53 signaling. Sci Adv (2021) 7(35). doi: 10.1126/sciadv.abf4304 PMC8386927 34433556

[49] Wang R Peng C Song J Hua Y Wu Q Deng L. Downregulated Rrs1 inhibits invasion and metastasis of Bt549 through Rpl11−C−Myc−Snail axis. Int J Oncol (2022) 60(3). doi: 10.3892/ijo.2022.5323 PMC8878750 35179222

[50] Xia W Liu Y Cheng T Xu T Dong M Hu X. Down-regulated lncrna Sbf2-As1 inhibits tumorigenesis and progression of breast cancer by sponging microrna-143 and repressing Rrs1. J Exp Clin Cancer research: CR (2020) 39(1):18. doi: 10.1186/s13046-020-1520-5 31952549 PMC6969426

[51] Zhang X Liu C Cao Y Liu L Sun F Hou L. Rrs1 knockdown inhibits the proliferation of neuroblastoma cell *Via* Pi3k/Akt/Nf-κb pathway. Pediatr Res (2022). doi: 10.1038/s41390-022-02073-0 35523884

[52] Yan X Wu S Liu Q Zhang J. Rrs1 promotes retinoblastoma cell proliferation and invasion *Via* activating the Akt/Mtor signaling pathway. BioMed Res Int (2020) 2020:2420437. doi: 10.1155/2020/2420437 33204686 PMC7652605

[53] Chen F Jin Y Feng L Zhang J Tai J Shi J. Rrs1 gene expression involved in the progression of papillary thyroid carcinoma. Cancer Cell Int (2018) 18:20. doi: 10.1186/s12935-018-0519-x 29449788 PMC5812111

[54] Hua Y Song J Peng C Wang R Ma Z Zhang J. Advances in the relationship between regulator of ribosome synthesis 1 (Rrs1) and diseases. Front Cell Dev Biol (2021) 9:620925. doi: 10.3389/fcell.2021.620925 33718361 PMC7947238

[55] Palmeri M Mehnert J Silk AW Jabbour SK Ganesan S Popli P. Real-world application of tumor mutational burden-high (Tmb-high) and microsatellite instability (Msi) confirms their utility as immunotherapy biomarkers. ESMO Open (2022) 7(1):100336. doi: 10.1016/j.esmoop.2021.100336 34953399 PMC8717431

