# Supplementary material for: Integrated bioinformatics and machine-learning screening for immune-related genes in diagnosing non-alcoholic fatty liver disease with ischemic stroke and RRS1 pan-cancer analysis
Source: Front Immunol. 2023 Apr 5;14:1113634. doi: 10.3389/fimmu.2023.1113634 (PMC10115222; doi:10.3389/fimmu.2023.1113634)
Supplement: Supplementary file 1 [file Table_1.docx]

| **Supplementary Table S1.** Differentially expressed genes in NAFLD. | | | | | | |
| --- | --- | --- | --- | --- | --- | --- |
| Gene | logFC | AveExpr | t | *P*.Value | adj.*P*.Val | B |
| SOCS2 | -1.07752 | 9.203124 | -8.32848 | 7.05E-14 | 5.65E-10 | 21.01652 |
| TMEM63A | 0.610709 | 10.15789 | 8.156204 | 1.85E-13 | 9.90E-10 | 20.09214 |
| P4HA1 | -1.05534 | 9.790012 | -7.94689 | 5.95E-13 | 2.38E-09 | 18.97768 |
| NAMPT | -0.63892 | 11.85102 | -7.61704 | 3.65E-12 | 9.75E-09 | 17.2426 |
| KIAA0040 | -0.88861 | 8.201377 | -7.58113 | 4.44E-12 | 1.02E-08 | 17.0554 |
| NFIL3 | -0.75112 | 10.33528 | -7.49502 | 7.09E-12 | 1.26E-08 | 16.60792 |
| IGFBP2 | -1.07477 | 10.7922 | -7.32677 | 1.76E-11 | 1.84E-08 | 15.73963 |
| CYP7A1 | 1.776031 | 10.26404 | 7.321141 | 1.81E-11 | 1.84E-08 | 15.71072 |
| LRRC31 | 0.688226 | 8.279545 | 7.28875 | 2.16E-11 | 1.84E-08 | 15.54456 |
| FBXO2 | 0.708282 | 8.585556 | 7.286536 | 2.18E-11 | 1.84E-08 | 15.53322 |
| LGALS4 | 0.995662 | 9.60375 | 7.117038 | 5.40E-11 | 3.43E-08 | 14.66907 |
| TNIK | -0.60826 | 8.250639 | -7.08912 | 6.26E-11 | 3.51E-08 | 14.5276 |
| SLITRK3 | -1.41903 | 8.166723 | -7.08672 | 6.34E-11 | 3.51E-08 | 14.51544 |
| INHBE | 0.6609 | 11.08876 | 7.031533 | 8.49E-11 | 4.25E-08 | 14.23657 |
| EPHA2 | -0.78569 | 8.303995 | -6.93781 | 1.39E-10 | 6.56E-08 | 13.76529 |
| RRS1 | -0.76082 | 8.686154 | -6.84313 | 2.28E-10 | 9.64E-08 | 13.29216 |
| RAB26 | 0.633444 | 9.971955 | 6.830457 | 2.44E-10 | 9.78E-08 | 13.22908 |
| DGCR5 | 0.61933 | 8.468614 | 6.802178 | 2.83E-10 | 1.10E-07 | 13.0885 |
| ISM1 | 0.689936 | 7.966278 | 6.798332 | 2.89E-10 | 1.10E-07 | 13.0694 |
| ME1 | 0.744175 | 9.03869 | 6.785332 | 3.09E-10 | 1.15E-07 | 13.00489 |
| PEG10 | 1.135009 | 8.638027 | 6.729287 | 4.13E-10 | 1.41E-07 | 12.72745 |
| MAMDC4 | 0.745105 | 9.405932 | 6.643736 | 6.42E-10 | 1.98E-07 | 12.30615 |
| FMO1 | 1.035526 | 6.737016 | 6.633279 | 6.78E-10 | 2.05E-07 | 12.25484 |
| HIVEP1 | -0.60834 | 9.802555 | -6.59538 | 8.23E-10 | 2.36E-07 | 12.06919 |
| SEC14L3 | 0.618469 | 6.943024 | 6.565925 | 9.58E-10 | 2.65E-07 | 11.92531 |
| ASPM | 0.700342 | 6.741601 | 6.541453 | 1.09E-09 | 2.95E-07 | 11.80601 |
| FNDC5 | 0.753383 | 10.14344 | 6.52099 | 1.20E-09 | 3.22E-07 | 11.70642 |
| GRIA3 | 0.650452 | 6.997966 | 6.472207 | 1.54E-09 | 3.93E-07 | 11.46967 |
| MCM2 | 0.619499 | 7.382968 | 6.444533 | 1.78E-09 | 4.36E-07 | 11.33578 |
| FGF14 | -0.68782 | 6.939099 | -6.42377 | 1.98E-09 | 4.59E-07 | 11.23551 |
| GINS2 | 0.868076 | 8.378289 | 6.405433 | 2.17E-09 | 4.76E-07 | 11.14712 |
| PNRC1 | -0.75968 | 9.60497 | -6.36507 | 2.66E-09 | 5.19E-07 | 10.95298 |
| RAPGEFL1 | 0.723445 | 7.395334 | 6.338115 | 3.04E-09 | 5.88E-07 | 10.82372 |
| RNF43 | 0.736969 | 8.792438 | 6.330843 | 3.16E-09 | 6.02E-07 | 10.7889 |
| CEBPA | 0.765962 | 11.11305 | 6.323394 | 3.28E-09 | 6.18E-07 | 10.75325 |
| PRAP1 | 0.610324 | 9.416014 | 6.256727 | 4.58E-09 | 7.97E-07 | 10.4352 |
| IL6 | -1.12217 | 6.237015 | -6.22696 | 5.31E-09 | 8.60E-07 | 10.29376 |
| PHLDA1 | -0.92073 | 8.969944 | -6.16364 | 7.27E-09 | 1.07E-06 | 9.994178 |
| FOSL1 | -0.79537 | 7.351184 | -6.15037 | 7.77E-09 | 1.11E-06 | 9.931642 |
| KLF5 | -0.62551 | 7.837852 | -6.13522 | 8.37E-09 | 1.18E-06 | 9.860307 |
| FABP4 | 0.838717 | 7.341363 | 6.121424 | 8.96E-09 | 1.23E-06 | 9.795407 |
| ELFN1 | 0.636822 | 9.321586 | 6.105607 | 9.69E-09 | 1.32E-06 | 9.721124 |
| FADS2 | 0.948009 | 10.74403 | 6.045989 | 1.30E-08 | 1.71E-06 | 9.442117 |
| FADS1 | 0.861684 | 10.93596 | 6.038583 | 1.35E-08 | 1.74E-06 | 9.407562 |
| PIM1 | -0.77829 | 9.689734 | -6.02938 | 1.41E-08 | 1.79E-06 | 9.364661 |
| VIL1 | -0.99009 | 8.579591 | -6.00545 | 1.59E-08 | 1.99E-06 | 9.253265 |
| WNT5A | 0.604147 | 7.709927 | 5.952425 | 2.05E-08 | 2.45E-06 | 9.0074 |
| AVPR1A | -1.00445 | 9.50038 | -5.95119 | 2.07E-08 | 2.45E-06 | 9.001681 |
| FAM107A | -0.69604 | 8.52282 | -5.94865 | 2.09E-08 | 2.45E-06 | 8.989936 |
| CEBPD | -0.60323 | 11.15405 | -5.94034 | 2.18E-08 | 2.53E-06 | 8.95152 |
| PNPLA3 | 0.612893 | 9.818352 | 5.847865 | 3.41E-08 | 3.48E-06 | 8.526269 |
| KCNK1 | -0.69294 | 8.307353 | -5.83156 | 3.69E-08 | 3.69E-06 | 8.451682 |
| IL1RN | -0.64 | 9.130383 | -5.78453 | 4.62E-08 | 4.43E-06 | 8.237268 |
| ACTG2 | -0.94617 | 7.667946 | -5.76164 | 5.15E-08 | 4.86E-06 | 8.133314 |
| TYMS | 0.73043 | 7.922419 | 5.751549 | 5.41E-08 | 5.01E-06 | 8.087552 |
| NNMT | -0.61486 | 12.10155 | -5.73618 | 5.82E-08 | 5.32E-06 | 8.017972 |
| GADD45G | -1.23238 | 9.736447 | -5.73047 | 5.98E-08 | 5.36E-06 | 7.992114 |
| SOCS1 | -0.74098 | 7.975108 | -5.66716 | 8.08E-08 | 6.61E-06 | 7.706772 |
| IP6K3 | 0.639405 | 6.965651 | 5.663051 | 8.24E-08 | 6.70E-06 | 7.68833 |
| SQLE | 0.704176 | 9.984008 | 5.661246 | 8.31E-08 | 6.71E-06 | 7.680225 |
| ENO3 | 0.699586 | 10.39655 | 5.659846 | 8.36E-08 | 6.71E-06 | 7.673943 |
| RNF152 | -0.62663 | 10.14788 | -5.64327 | 9.05E-08 | 7.07E-06 | 7.599616 |
| ADAMTS1 | -0.9739 | 8.66413 | -5.60779 | 1.07E-07 | 8.32E-06 | 7.440973 |
| RND1 | -0.87935 | 9.146619 | -5.52119 | 1.60E-07 | 1.17E-05 | 7.056357 |
| FILIP1L | -0.65932 | 8.076773 | -5.48757 | 1.88E-07 | 1.31E-05 | 6.90805 |
| FOSB | -1.74733 | 8.050834 | -5.46242 | 2.11E-07 | 1.45E-05 | 6.797472 |
| NAT8B | 0.697357 | 8.590839 | 5.420714 | 2.56E-07 | 1.67E-05 | 6.614855 |
| CXCL9 | 0.745319 | 7.73 | 5.418408 | 2.59E-07 | 1.68E-05 | 6.604784 |
| MYC | -1.03139 | 8.824864 | -5.39393 | 2.89E-07 | 1.83E-05 | 6.498026 |
| CRISPLD2 | -0.65738 | 9.096504 | -5.35421 | 3.47E-07 | 2.10E-05 | 6.325505 |
| CISH | -0.72828 | 9.424872 | -5.25267 | 5.51E-07 | 2.97E-05 | 5.888145 |
| APOLD1 | -0.87783 | 7.625547 | -5.18314 | 7.55E-07 | 3.76E-05 | 5.591877 |
| PPP1R15A | -0.64915 | 8.848284 | -5.14472 | 8.96E-07 | 4.18E-05 | 5.429274 |
| KRT222 | 0.651021 | 6.933931 | 5.124644 | 9.80E-07 | 4.49E-05 | 5.344639 |
| TMEM45B | 0.619344 | 10.38185 | 5.047094 | 1.38E-06 | 5.83E-05 | 5.019795 |
| PTX3 | -0.70495 | 5.878901 | -5.02563 | 1.52E-06 | 6.20E-05 | 4.9305 |
| DBP | 0.721832 | 8.391465 | 5.013674 | 1.60E-06 | 6.44E-05 | 4.880848 |
| IL1RL1 | -0.71518 | 6.312016 | -4.98009 | 1.86E-06 | 7.19E-05 | 4.741859 |
| CRYAA | 0.670571 | 9.016233 | 4.973743 | 1.91E-06 | 7.34E-05 | 4.715655 |
| CD274 | -0.60894 | 7.520592 | -4.91395 | 2.48E-06 | 9.09E-05 | 4.469994 |
| FOSL2 | -0.68064 | 9.054121 | -4.88756 | 2.78E-06 | 9.94E-05 | 4.362221 |
| GADD45B | -0.62632 | 10.46814 | -4.8708 | 2.99E-06 | 0.000105 | 4.294007 |
| OXT | 0.641031 | 9.062092 | 4.741025 | 5.21E-06 | 0.000161 | 3.771279 |
| CDH15 | 0.748612 | 6.987686 | 4.676182 | 6.85E-06 | 0.000196 | 3.513866 |
| CNN1 | -0.63915 | 7.115322 | -4.64282 | 7.89E-06 | 0.000217 | 3.38244 |
| ZC3H12A | -0.69409 | 7.938715 | -4.64177 | 7.92E-06 | 0.000217 | 3.37829 |
| SOCS3 | -0.7097 | 7.516415 | -4.58854 | 9.89E-06 | 0.000255 | 3.170002 |
| IER3 | -0.74051 | 8.886 | -4.58814 | 9.91E-06 | 0.000255 | 3.168443 |
| SPSB1 | -0.67545 | 8.555068 | -4.56674 | 1.08E-05 | 0.000274 | 3.085212 |
| NR4A1 | -0.74535 | 7.588528 | -4.52395 | 1.29E-05 | 0.00031 | 2.919646 |
| VNN3 | -0.60052 | 8.783459 | -4.51482 | 1.34E-05 | 0.000317 | 2.884475 |
| TRHDE | 0.628219 | 7.307761 | 4.496023 | 1.45E-05 | 0.000337 | 2.812198 |
| SIK1 | -0.73964 | 8.102507 | -4.35788 | 2.54E-05 | 0.000524 | 2.287985 |
| KLF6 | -0.61833 | 9.522681 | -4.35403 | 2.58E-05 | 0.00053 | 2.273569 |
| SLC7A1 | -0.62442 | 6.974902 | -4.317 | 2.99E-05 | 0.000595 | 2.135236 |
| JUNB | -0.90698 | 9.389681 | -4.26555 | 3.67E-05 | 0.000691 | 1.944482 |
| NR4A2 | -0.82616 | 7.702878 | -4.24381 | 4.00E-05 | 0.000726 | 1.864442 |
| EEF1A2 | 0.681244 | 6.465984 | 4.236711 | 4.11E-05 | 0.000738 | 1.838353 |
| THBS1 | -0.69981 | 10.52597 | -4.23378 | 4.16E-05 | 0.000742 | 1.827589 |
| TGFB3 | -0.64555 | 8.747101 | -4.18689 | 5.00E-05 | 0.000858 | 1.656293 |
| RTP3 | 0.623761 | 9.925421 | 4.162393 | 5.50E-05 | 0.000912 | 1.567369 |
| RGS2 | -0.61533 | 9.098575 | -4.159 | 5.57E-05 | 0.000922 | 1.555074 |
| RASD1 | -0.82021 | 8.050971 | -4.15622 | 5.63E-05 | 0.000927 | 1.545043 |
| IGFBP1 | -1.03816 | 10.98689 | -4.04514 | 8.64E-05 | 0.001279 | 1.147392 |
| FOS | -1.27245 | 9.935256 | -4.01982 | 9.51E-05 | 0.001377 | 1.057915 |
| ZFP36 | -0.60889 | 8.781267 | -3.94103 | 0.000128 | 0.001695 | 0.782392 |
| HBEGF | -0.64092 | 7.946817 | -3.9251 | 0.000136 | 0.001773 | 0.727195 |
| PTGS2 | -0.63727 | 7.975263 | -3.90145 | 0.000148 | 0.001897 | 0.645607 |
| NPY6R | 0.744984 | 7.988529 | 3.864106 | 0.000171 | 0.002109 | 0.517547 |
| CCL2 | -0.67222 | 10.03763 | -3.83771 | 0.000188 | 0.002263 | 0.427646 |
| MIR21 | -0.73135 | 9.308769 | -3.81539 | 0.000204 | 0.00241 | 0.351981 |
| AKR1B10 | 1.110785 | 6.97117 | 3.795358 | 0.00022 | 0.002553 | 0.284395 |
| EMP1 | -0.67817 | 8.599122 | -3.78874 | 0.000225 | 0.002605 | 0.262132 |
| DEFA3 | 0.620824 | 7.97678 | 3.740401 | 0.000268 | 0.002963 | 0.100445 |
| NCAM2 | 0.656533 | 9.307224 | 3.585441 | 0.000465 | 0.004465 | -0.40642 |
| SAA1 | -0.67988 | 8.554786 | -3.25124 | 0.001442 | 0.010443 | -1.43853 |
| EGR1 | -0.8395 | 11.11282 | -3.22474 | 0.001572 | 0.011104 | -1.51668 |

| **Supplementary Table S2.** Differentially expressed genes in IS. | | | | | | |
| --- | --- | --- | --- | --- | --- | --- |
| Gene | logFC | AveExpr | t | *P*.Value | adj.*P*.Val | B |
| C19orf24 | -0.72259 | 0.650731 | -12.0583 | 3.53E-24 | 4.85E-21 | 44.30545 |
| CPD | 0.831794 | 1.228017 | 12.01544 | 4.63E-24 | 4.85E-21 | 44.03901 |
| SLC22A4 | 0.8691 | 1.742327 | 11.77588 | 2.11E-23 | 1.54E-20 | 42.55109 |
| PLXDC2 | 0.674716 | 0.568066 | 11.7087 | 3.23E-23 | 1.93E-20 | 42.13413 |
| SRPK1 | 0.779309 | 1.117986 | 11.38403 | 2.51E-22 | 1.31E-19 | 40.12095 |
| RRS1 | -0.64 | 0.419939 | -11.2964 | 4.37E-22 | 2.00E-19 | 39.57828 |
| TMC6 | -0.71957 | 0.286019 | -10.9272 | 4.47E-21 | 1.48E-18 | 37.29692 |
| MRPL41 | -0.6971 | 0.517783 | -10.9232 | 4.58E-21 | 1.48E-18 | 37.2726 |
| FLT3LG | -0.91846 | 0.769236 | -10.8899 | 5.65E-21 | 1.69E-18 | 37.06702 |
| CLEC4D | 1.075581 | 1.15995 | 10.44763 | 9.02E-20 | 2.36E-17 | 34.34866 |
| EVL | -0.66746 | 0.968775 | -10.2096 | 3.98E-19 | 8.32E-17 | 32.89327 |
| KCNJ15 | 0.805287 | 1.337088 | 10.16526 | 5.24E-19 | 1.04E-16 | 32.62303 |
| AES | -0.6707 | 1.446617 | -10.0953 | 8.09E-19 | 1.54E-16 | 32.19684 |
| APRT | -0.67915 | 1.806796 | -10.0298 | 1.21E-18 | 2.03E-16 | 31.79852 |
| CD163 | 0.896881 | 0.922846 | 9.995725 | 1.50E-18 | 2.41E-16 | 31.59147 |
| DHRS3 | -0.69863 | 0.634651 | -9.91223 | 2.51E-18 | 3.90E-16 | 31.08477 |
| LEF1 | -0.8079 | 0.274245 | -9.84373 | 3.84E-18 | 5.35E-16 | 30.66981 |
| NFIL3 | 0.632263 | 2.150132 | 9.752159 | 6.75E-18 | 8.56E-16 | 30.11608 |
| S100A12 | 0.884088 | 2.854844 | 9.746724 | 6.98E-18 | 8.59E-16 | 30.08326 |
| TXN | 0.700763 | 1.938763 | 9.687856 | 1.00E-17 | 1.13E-15 | 29.728 |
| GYG1 | 0.624438 | 1.897876 | 9.671341 | 1.11E-17 | 1.22E-15 | 29.62842 |
| LHFPL2 | 0.744917 | 0.934941 | 9.628469 | 1.44E-17 | 1.47E-15 | 29.37013 |
| F5 | 0.776058 | 1.023009 | 9.578751 | 1.96E-17 | 1.91E-15 | 29.07097 |
| ICAM2 | -0.62431 | 1.503945 | -9.49829 | 3.21E-17 | 2.98E-15 | 28.58762 |
| DIRC2 | 0.637996 | 0.192792 | 9.472488 | 3.76E-17 | 3.42E-15 | 28.43288 |
| ZNF438 | 0.705783 | 0.025578 | 9.37011 | 7.02E-17 | 6.25E-15 | 27.81994 |
| IRAK3 | 0.792937 | 1.072611 | 9.337297 | 8.58E-17 | 7.48E-15 | 27.62387 |
| CLEC4E | 0.771137 | 1.780608 | 9.332952 | 8.81E-17 | 7.52E-15 | 27.59792 |
| MBOAT2 | 0.722696 | 0.959733 | 9.300088 | 1.08E-16 | 8.83E-15 | 27.40177 |
| MCEMP1 | 1.283738 | 1.646607 | 9.280229 | 1.21E-16 | 9.59E-15 | 27.28333 |
| KIF1B | 0.633748 | 0.175203 | 9.246261 | 1.49E-16 | 1.12E-14 | 27.08092 |
| CR1 | 0.625007 | 0.360595 | 9.157801 | 2.55E-16 | 1.76E-14 | 26.55481 |
| BAZ2B | 0.689099 | 2.172578 | 9.101646 | 3.59E-16 | 2.34E-14 | 26.22161 |
| LIN7A | 0.725434 | 0.299403 | 9.062918 | 4.53E-16 | 2.83E-14 | 25.99218 |
| CCR7 | -1.16834 | 0.918714 | -9.03237 | 5.45E-16 | 3.31E-14 | 25.81141 |
| GOLGA8A | -0.69202 | 1.337389 | -8.77541 | 2.55E-15 | 1.35E-13 | 24.29872 |
| ORMDL3 | -0.70061 | 0.60078 | -8.72496 | 3.45E-15 | 1.75E-13 | 24.0034 |
| ITM2C | -0.73309 | 0.341513 | -8.72414 | 3.47E-15 | 1.75E-13 | 23.9986 |
| FGD4 | 0.612312 | 0.052834 | 8.687535 | 4.31E-15 | 2.12E-13 | 23.78471 |
| SLC26A8 | 0.950542 | 0.375081 | 8.678164 | 4.56E-15 | 2.19E-13 | 23.73 |
| PLEKHA1 | -0.66847 | 0.137578 | -8.55327 | 9.59E-15 | 4.22E-13 | 23.00289 |
| HAPLN3 | -0.6111 | 0.253879 | -8.53128 | 1.09E-14 | 4.66E-13 | 22.87523 |
| SPOCK2 | -0.6205 | 0.822562 | -8.49243 | 1.37E-14 | 5.70E-13 | 22.65003 |
| HSDL2 | 0.615932 | 0.015324 | 8.409329 | 2.25E-14 | 8.95E-13 | 22.16959 |
| ACSL1 | 0.664106 | 2.638649 | 8.21729 | 6.94E-14 | 2.44E-12 | 21.06621 |
| VNN3 | 0.639193 | 0.561579 | 8.143451 | 1.07E-13 | 3.54E-12 | 20.64462 |
| SLC22A15 | 0.602009 | 0.559204 | 8.13687 | 1.11E-13 | 3.60E-12 | 20.60712 |
| IL7R | -0.73844 | 1.953316 | -8.12992 | 1.15E-13 | 3.69E-12 | 20.56755 |
| NCR3 | -0.66881 | 0.239761 | -8.10601 | 1.33E-13 | 4.14E-12 | 20.43143 |
| HAL | 0.615895 | 0.687601 | 7.992994 | 2.56E-13 | 7.43E-12 | 19.79037 |
| TLR5 | 0.807226 | 1.153604 | 7.852328 | 5.75E-13 | 1.48E-11 | 18.99776 |
| PRRG4 | 0.753934 | 0.222204 | 7.844553 | 6.01E-13 | 1.53E-11 | 18.95413 |
| DYSF | 0.658704 | 2.378003 | 7.836352 | 6.30E-13 | 1.60E-11 | 18.90812 |
| IL18RAP | 0.837801 | 2.390564 | 7.824746 | 6.74E-13 | 1.68E-11 | 18.84305 |
| TMEM88 | 0.605197 | 0.298148 | 7.782363 | 8.59E-13 | 2.09E-11 | 18.60579 |
| MMP9 | 1.187385 | 1.779132 | 7.750597 | 1.03E-12 | 2.39E-11 | 18.42834 |
| IL2RB | -0.7411 | 1.112917 | -7.73737 | 1.11E-12 | 2.57E-11 | 18.35453 |
| NELL2 | -0.99042 | 1.169885 | -7.72816 | 1.17E-12 | 2.69E-11 | 18.30318 |
| ANXA3 | 0.884526 | 1.955788 | 7.722075 | 1.21E-12 | 2.74E-11 | 18.26927 |
| FAIM3 | -0.69243 | 1.431956 | -7.60601 | 2.34E-12 | 4.98E-11 | 17.62475 |
| CD79A | -0.75745 | 0.59621 | -7.50944 | 4.04E-12 | 8.12E-11 | 17.09188 |
| GPR97 | 0.664974 | 1.148359 | 7.40454 | 7.29E-12 | 1.31E-10 | 16.51669 |
| GZMM | -0.62776 | 0.179042 | -7.23259 | 1.90E-11 | 3.11E-10 | 15.58231 |
| FCGR1A | 0.77663 | 1.247149 | 7.225223 | 1.98E-11 | 3.22E-10 | 15.54253 |
| NDUFB3 | 0.668559 | 1.486031 | 7.122813 | 3.48E-11 | 5.18E-10 | 14.99152 |
| SIPA1L2 | 0.696431 | 0.374156 | 7.068253 | 4.70E-11 | 6.77E-10 | 14.6996 |
| CLEC5A | 0.70447 | 0.209466 | 6.93753 | 9.58E-11 | 1.29E-09 | 14.0049 |
| TCL1A | -0.78366 | 0.837488 | -6.927 | 1.01E-10 | 1.36E-09 | 13.94922 |
| TRIB2 | -0.62578 | 0.388057 | -6.90453 | 1.15E-10 | 1.52E-09 | 13.83064 |
| LY96 | 0.970681 | 2.05361 | 6.875829 | 1.34E-10 | 1.75E-09 | 13.67939 |
| BCL2A1 | 0.824199 | 2.250217 | 6.861838 | 1.44E-10 | 1.85E-09 | 13.60579 |
| LILRA5 | 0.692246 | 0.295644 | 6.753842 | 2.58E-10 | 3.08E-09 | 13.04043 |
| CYP1B1 | 0.655258 | 0.381834 | 6.69018 | 3.62E-10 | 4.24E-09 | 12.70946 |
| FCRL3 | -0.61689 | 0.302077 | -6.57923 | 6.53E-10 | 7.31E-09 | 12.13682 |
| LRG1 | 0.633672 | 1.582806 | 6.505494 | 9.63E-10 | 1.04E-08 | 11.75926 |
| CD247 | -0.61873 | 1.176185 | -6.5014 | 9.84E-10 | 1.06E-08 | 11.73835 |
| CASP5 | 0.774869 | 0.633862 | 6.480539 | 1.10E-09 | 1.15E-08 | 11.63203 |
| MAL | -0.714 | 1.443003 | -6.45887 | 1.23E-09 | 1.27E-08 | 11.52177 |
| C12orf57 | -0.66462 | 0.746141 | -6.38483 | 1.81E-09 | 1.80E-08 | 11.14672 |
| TPST1 | 0.890953 | 0.242614 | 6.279327 | 3.12E-09 | 2.89E-08 | 10.61663 |
| NOG | -0.80179 | 0.54703 | -6.20421 | 4.59E-09 | 4.09E-08 | 10.24245 |
| ECHDC3 | 0.983583 | 0.368886 | 6.184269 | 5.08E-09 | 4.47E-08 | 10.14356 |
| FOLR3 | 0.996129 | 1.846219 | 6.106778 | 7.54E-09 | 6.26E-08 | 9.761162 |
| PTGS2 | 0.617071 | 0.460584 | 6.024636 | 1.14E-08 | 9.17E-08 | 9.35906 |
| ZNF467 | 0.624079 | 1.383308 | 5.974571 | 1.47E-08 | 1.15E-07 | 9.115645 |
| PGLYRP1 | 0.702414 | 1.114774 | 5.943804 | 1.71E-08 | 1.32E-07 | 8.966691 |
| TCN1 | 0.713539 | 0.436344 | 5.879571 | 2.36E-08 | 1.74E-07 | 8.657284 |
| CA4 | 0.731596 | 0.444617 | 5.499245 | 1.50E-07 | 9.28E-07 | 6.870334 |
| PRSS33 | -1.08902 | 0.630112 | -5.48854 | 1.57E-07 | 9.65E-07 | 6.821196 |
| GZMK | -0.714 | 1.704389 | -5.41915 | 2.19E-07 | 1.28E-06 | 6.504192 |
| FKBP5 | 0.608467 | 1.673482 | 5.304301 | 3.75E-07 | 2.08E-06 | 5.985665 |
| PDZK1IP1 | -0.75121 | 1.92408 | -4.59183 | 8.90E-06 | 3.58E-05 | 2.948783 |
| HP | 0.72651 | 0.885576 | 4.104937 | 6.45E-05 | 0.000208 | 1.066508 |
| CEACAM8 | 0.684929 | 1.118378 | 3.365548 | 0.000958 | 0.002363 | -1.45906 |
| ORM1 | 0.737541 | 1.146924 | 3.322829 | 0.001106 | 0.002686 | -1.59194 |
| DEFA4 | 0.739667 | 1.55552 | 3.293985 | 0.001218 | 0.00293 | -1.68084 |
| S100P | 0.616001 | 2.472313 | 3.087653 | 0.002382 | 0.005336 | -2.29688 |
| OLFM4 | 0.607731 | 0.38512 | 2.302409 | 0.022612 | 0.038656 | -4.31063 |

| **Supplementary Table S3.** The correlation analysis between candidate genes and immune cells. | | | |
| --- | --- | --- | --- |
| Immune Cell | Gene | Correlation | *P* value |
| Activated.B.cell | RRS1 | 0.122552802 | 0.206056142 |
| Activated.B.cell | S100A12 | -0.15412574 | 0.111246473 |
| Activated.B.cell | VNN3 | -0.152181162 | 0.115799141 |
| Activated.B.cell | MMP9 | -0.169025356 | 0.080337894 |
| Activated.B.cell | FCGR1A | -0.050663663 | 0.602561674 |
| Activated.B.cell | PTGS2 | -0.002510319 | 0.979429248 |
| Activated.CD4.T.cell | RRS1 | 0.284937171 | 0.002894279 |
| Activated.CD4.T.cell | S100A12 | -0.209907301 | 0.02922857 |
| Activated.CD4.T.cell | VNN3 | -0.364009641 | 0.000119673 |
| Activated.CD4.T.cell | MMP9 | -0.485774033 | 9.87E-08 |
| Activated.CD4.T.cell | FCGR1A | 0.003786913 | 0.968972544 |
| Activated.CD4.T.cell | PTGS2 | 0.12906468 | 0.183107608 |
| Activated.CD8.T.cell | RRS1 | 0.258843255 | 0.00697234 |
| Activated.CD8.T.cell | S100A12 | -0.256838124 | 0.00729104 |
| Activated.CD8.T.cell | VNN3 | -0.374108053 | 7.50E-05 |
| Activated.CD8.T.cell | MMP9 | -0.389781502 | 3.05E-05 |
| Activated.CD8.T.cell | FCGR1A | 0.02862335 | 0.768710903 |
| Activated.CD8.T.cell | PTGS2 | -0.032334049 | 0.739736638 |
| Activated.dendritic.cell | RRS1 | -0.254203702 | 0.008084297 |
| Activated.dendritic.cell | S100A12 | 0.528038452 | 4.28E-09 |
| Activated.dendritic.cell | VNN3 | 0.379528804 | 5.80E-05 |
| Activated.dendritic.cell | MMP9 | 0.517517494 | 9.74E-09 |
| Activated.dendritic.cell | FCGR1A | 0.090928794 | 0.349322276 |
| Activated.dendritic.cell | PTGS2 | 0.044294979 | 0.648969669 |
| CD56bright.natural.killer.cell | RRS1 | 0.163260834 | 0.091354836 |
| CD56bright.natural.killer.cell | S100A12 | -0.128902566 | 0.183662894 |
| CD56bright.natural.killer.cell | VNN3 | -0.116874827 | 0.227977703 |
| CD56bright.natural.killer.cell | MMP9 | 0.093286906 | 0.336913731 |
| CD56bright.natural.killer.cell | FCGR1A | 0.040112702 | 0.68020845 |
| CD56bright.natural.killer.cell | PTGS2 | -0.503245075 | 2.84E-08 |
| CD56dim.natural.killer.cell | RRS1 | 0.072146484 | 0.457487704 |
| CD56dim.natural.killer.cell | S100A12 | 0.132708669 | 0.170947672 |
| CD56dim.natural.killer.cell | VNN3 | 0.130202826 | 0.178975309 |
| CD56dim.natural.killer.cell | MMP9 | 0.35102628 | 0.000195487 |
| CD56dim.natural.killer.cell | FCGR1A | -0.067921508 | 0.484892883 |
| CD56dim.natural.killer.cell | PTGS2 | -0.291416091 | 0.002213747 |
| Eosinophil | RRS1 | -0.312183829 | 0.001058163 |
| Eosinophil | S100A12 | 0.254246735 | 0.007925047 |
| Eosinophil | VNN3 | 0.140777578 | 0.145982589 |
| Eosinophil | MMP9 | 0.209628786 | 0.029448439 |
| Eosinophil | FCGR1A | 0.100017386 | 0.303056235 |
| Eosinophil | PTGS2 | 0.412678301 | 9.07E-06 |
| Gamma.delta.T.cell | RRS1 | -0.087341736 | 0.368173657 |
| Gamma.delta.T.cell | S100A12 | 0.280051066 | 0.003330235 |
| Gamma.delta.T.cell | VNN3 | 0.043146894 | 0.657012462 |
| Gamma.delta.T.cell | MMP9 | -0.078863256 | 0.417193996 |
| Gamma.delta.T.cell | FCGR1A | 0.297275089 | 0.001781825 |
| Gamma.delta.T.cell | PTGS2 | 0.098112021 | 0.3124065 |
| Immature.B.cell | RRS1 | -0.260805777 | 0.006544404 |
| Immature.B.cell | S100A12 | 0.119851757 | 0.216643861 |
| Immature.B.cell | VNN3 | 0.33957339 | 0.000349807 |
| Immature.B.cell | MMP9 | 0.236327781 | 0.013801124 |
| Immature.B.cell | FCGR1A | 0.157349827 | 0.103875701 |
| Immature.B.cell | PTGS2 | -0.078239061 | 0.420901755 |
| Immature.dendritic.cell | RRS1 | -0.011279736 | 0.907644568 |
| Immature.dendritic.cell | S100A12 | 0.098653811 | 0.309728893 |
| Immature.dendritic.cell | VNN3 | 0.184010213 | 0.056710615 |
| Immature.dendritic.cell | MMP9 | 0.608994298 | 2.69E-12 |
| Immature.dendritic.cell | FCGR1A | -0.068274001 | 0.482624328 |
| Immature.dendritic.cell | PTGS2 | -0.238975676 | 0.012745186 |
| MDSC | RRS1 | 0.244572104 | 0.010905253 |
| MDSC | S100A12 | 0.083920048 | 0.387863114 |
| MDSC | VNN3 | 0.034620405 | 0.721662357 |
| MDSC | MMP9 | 0.14573221 | 0.132345369 |
| MDSC | FCGR1A | 0.358446842 | 0.000139459 |
| MDSC | PTGS2 | 0.030905025 | 0.750854335 |
| Macrophage | RRS1 | -0.131870016 | 0.173437992 |
| Macrophage | S100A12 | 0.239574898 | 0.012516249 |
| Macrophage | VNN3 | 0.469518992 | 4.14E-07 |
| Macrophage | MMP9 | 0.769769403 | 2.15E-22 |
| Macrophage | FCGR1A | -0.02463161 | 0.800238103 |
| Macrophage | PTGS2 | -0.123991645 | 0.20107144 |
| Mast.cell | RRS1 | -0.249106862 | 0.009484258 |
| Mast.cell | S100A12 | -0.037946705 | 0.696606469 |
| Mast.cell | VNN3 | 0.016852916 | 0.862369601 |
| Mast.cell | MMP9 | 0.067373876 | 0.488428573 |
| Mast.cell | FCGR1A | 0.063329578 | 0.514958622 |
| Mast.cell | PTGS2 | 0.402484596 | 1.57E-05 |
| Monocyte | RRS1 | 0.034658512 | 0.721368575 |
| Monocyte | S100A12 | 0.209897774 | 0.029236068 |
| Monocyte | VNN3 | 0.119056465 | 0.219368075 |
| Monocyte | MMP9 | 0.413484302 | 8.68E-06 |
| Monocyte | FCGR1A | 0.104785563 | 0.280468943 |
| Monocyte | PTGS2 | -0.177337109 | 0.066346132 |
| Natural.killer.T.cell | RRS1 | 0.017681748 | 0.855670798 |
| Natural.killer.T.cell | S100A12 | -0.269695036 | 0.004762993 |
| Natural.killer.T.cell | VNN3 | -0.198910134 | 0.039194999 |
| Natural.killer.T.cell | MMP9 | -0.155211425 | 0.108720302 |
| Natural.killer.T.cell | FCGR1A | 0.003701172 | 0.969674715 |
| Natural.killer.T.cell | PTGS2 | 0.009393451 | 0.923134748 |
| Natural.killer.cell | RRS1 | -0.163689541 | 0.090498655 |
| Natural.killer.cell | S100A12 | 0.565022913 | 1.89E-10 |
| Natural.killer.cell | VNN3 | 0.049920451 | 0.607377968 |
| Natural.killer.cell | MMP9 | 0.282980761 | 0.00300224 |
| Natural.killer.cell | FCGR1A | 0.377919675 | 5.52E-05 |
| Natural.killer.cell | PTGS2 | -0.036759259 | 0.705656475 |
| Neutrophil | RRS1 | -0.301075576 | 0.001612699 |
| Neutrophil | S100A12 | 0.329692368 | 0.000493341 |
| Neutrophil | VNN3 | 0.675621862 | 0 |
| Neutrophil | MMP9 | 0.512263436 | 1.45E-08 |
| Neutrophil | FCGR1A | 0.057627773 | 0.553576551 |
| Neutrophil | PTGS2 | 0.198134171 | 0.039827218 |
| Plasmacytoid.dendritic.cell | RRS1 | -0.11102537 | 0.252228728 |
| Plasmacytoid.dendritic.cell | S100A12 | 0.257176339 | 0.007211694 |
| Plasmacytoid.dendritic.cell | VNN3 | 0.387054979 | 4.03E-05 |
| Plasmacytoid.dendritic.cell | MMP9 | 0.206584958 | 0.031944155 |
| Plasmacytoid.dendritic.cell | FCGR1A | 0.01470942 | 0.879901426 |
| Plasmacytoid.dendritic.cell | PTGS2 | 0.49757185 | 4.29E-08 |
| Regulatory.T.cell | RRS1 | 0.201310888 | 0.036850489 |
| Regulatory.T.cell | S100A12 | 0.185508227 | 0.054587635 |
| Regulatory.T.cell | VNN3 | 0.096830432 | 0.31827932 |
| Regulatory.T.cell | MMP9 | -0.034163281 | 0.725583496 |
| Regulatory.T.cell | FCGR1A | 0.272438653 | 0.004337867 |
| Regulatory.T.cell | PTGS2 | 0.308916871 | 0.001142139 |
| T.follicular.helper.cell | RRS1 | 0.008621757 | 0.929351488 |
| T.follicular.helper.cell | S100A12 | 0.079594714 | 0.412873334 |
| T.follicular.helper.cell | VNN3 | 0.076195376 | 0.432583179 |
| T.follicular.helper.cell | MMP9 | 0.402252147 | 1.59E-05 |
| T.follicular.helper.cell | FCGR1A | 0.011827555 | 0.903303309 |
| T.follicular.helper.cell | PTGS2 | -0.276659037 | 0.003749907 |
| Type.1.T.helper.cell | RRS1 | -0.054674326 | 0.573565717 |
| Type.1.T.helper.cell | S100A12 | 0.068676581 | 0.480040391 |
| Type.1.T.helper.cell | VNN3 | 0.147836939 | 0.12666784 |
| Type.1.T.helper.cell | MMP9 | 0.246588197 | 0.01009212 |
| Type.1.T.helper.cell | FCGR1A | 0.146055775 | 0.131479098 |
| Type.1.T.helper.cell | PTGS2 | 0.023397886 | 0.810050076 |
| Type.17.T.helper.cell | RRS1 | -0.118103785 | 0.223098901 |
| Type.17.T.helper.cell | S100A12 | 0.095690863 | 0.324555343 |
| Type.17.T.helper.cell | VNN3 | 0.305858032 | 0.001347702 |
| Type.17.T.helper.cell | MMP9 | 0.305040179 | 0.001327115 |
| Type.17.T.helper.cell | FCGR1A | 0.049129843 | 0.613603581 |
| Type.17.T.helper.cell | PTGS2 | -0.170935082 | 0.076930391 |
| Type.2.T.helper.cell | RRS1 | 0.227585813 | 0.018024012 |
| Type.2.T.helper.cell | S100A12 | -0.200523042 | 0.037449242 |
| Type.2.T.helper.cell | VNN3 | -0.306315318 | 0.001324568 |
| Type.2.T.helper.cell | MMP9 | -0.498506666 | 4.01E-08 |
| Type.2.T.helper.cell | FCGR1A | 0.084945947 | 0.382067474 |
| Type.2.T.helper.cell | PTGS2 | 0.290048992 | 0.002327251 |
| Effector.memory.CD4.T.cell | RRS1 | 0.243095449 | 0.011406737 |
| Effector.memory.CD4.T.cell | S100A12 | -0.367939179 | 8.94E-05 |
| Effector.memory.CD4.T.cell | VNN3 | -0.407804358 | 1.41E-05 |
| Effector.memory.CD4.T.cell | MMP9 | -0.396216888 | 2.19E-05 |
| Effector.memory.CD4.T.cell | FCGR1A | -0.070484225 | 0.46853034 |
| Effector.memory.CD4.T.cell | PTGS2 | -0.039231471 | 0.686862502 |
| Memory.B.cell | RRS1 | 0.212390561 | 0.027503835 |
| Memory.B.cell | S100A12 | -0.264712327 | 0.005631 |
| Memory.B.cell | VNN3 | -0.368058533 | 9.94E-05 |
| Memory.B.cell | MMP9 | -0.382650655 | 4.37E-05 |
| Memory.B.cell | FCGR1A | -0.029523635 | 0.761650161 |
| Memory.B.cell | PTGS2 | 0.03258651 | 0.737777997 |
| Central.memory.CD4.T.cell | RRS1 | 0.2633018 | 0.006033967 |
| Central.memory.CD4.T.cell | S100A12 | -0.136933967 | 0.157606687 |
| Central.memory.CD4.T.cell | VNN3 | -0.544418722 | 1.72E-09 |
| Central.memory.CD4.T.cell | MMP9 | -0.527844598 | 4.35E-09 |
| Central.memory.CD4.T.cell | FCGR1A | -0.079415624 | 0.41392878 |
| Central.memory.CD4.T.cell | PTGS2 | 0.036859291 | 0.704892487 |
| Central.memory.CD8.T.cell | RRS1 | 0.277296674 | 0.003775562 |
| Central.memory.CD8.T.cell | S100A12 | -0.182440479 | 0.05878361 |
| Central.memory.CD8.T.cell | VNN3 | -0.05838978 | 0.54776997 |
| Central.memory.CD8.T.cell | MMP9 | -0.095944897 | 0.323266606 |
| Central.memory.CD8.T.cell | FCGR1A | -0.14518407 | 0.133822823 |
| Central.memory.CD8.T.cell | PTGS2 | 0.01131787 | 0.907451374 |
| Effector.memory.CD8.T.cell | RRS1 | 0.254603828 | 0.007982564 |
| Effector.memory.CD8.T.cell | S100A12 | -0.312757829 | 0.000982337 |
| Effector.memory.CD8.T.cell | VNN3 | -0.188211533 | 0.051219367 |
| Effector.memory.CD8.T.cell | MMP9 | -0.287748948 | 0.002530114 |
| Effector.memory.CD8.T.cell | FCGR1A | -0.059204462 | 0.542759326 |
| Effector.memory.CD8.T.cell | PTGS2 | 0.159331407 | 0.099538888 |
